# Multi-generation genomic prediction of maize yield using parametric and non-parametric sparse selection indices

**DOI:** 10.1038/s41437-021-00474-1

**Published:** 2021-09-25

**Authors:** Marco Lopez-Cruz, Yoseph Beyene, Manje Gowda, Jose Crossa, Paulino Pérez-Rodríguez, Gustavo de los Campos

**Affiliations:** 1 Department of Plant, Soil and Microbial Sciences, Michigan State University, East Lansing, MI USA; 2 Department of Epidemiology and Biostatistics, Michigan State University, East Lansing, MI USA; 3 Global Maize Program, International Maize and Wheat Improvement Center (CIMMYT), Nairobi, Kenya; 4 Biometrics and Statistics Unit, International Maize and Wheat Improvement Center (CIMMYT), Mexico, Mexico; 5 Colegio de Postgraduados, Montecillos, Edo. de México, Mexico; 6 Department of Statistics and Probability, Michigan State University, East Lansing, MI USA; 7 Institute for Quantitative Health Science and Engineering, Michigan State University, East Lansing, MI USA

**Keywords:** Quantitative trait, Genetic models

## Abstract

Genomic prediction models are often calibrated using multi-generation data. Over time, as data accumulates, training data sets become increasingly heterogeneous. Differences in allele frequency and linkage disequilibrium patterns between the training and prediction genotypes may limit prediction accuracy. This leads to the question of whether all available data or a subset of it should be used to calibrate genomic prediction models. Previous research on training set optimization has focused on identifying a subset of the available data that is optimal for a given prediction set. However, this approach does not contemplate the possibility that different training sets may be optimal for different prediction genotypes. To address this problem, we recently introduced a sparse selection index (SSI) that identifies an optimal training set for each individual in a prediction set. Using additive genomic relationships, the SSI can provide increased accuracy relative to genomic-BLUP (GBLUP). Non-parametric genomic models using Gaussian kernels (KBLUP) have, in some cases, yielded higher prediction accuracies than standard additive models. Therefore, here we studied whether combining SSIs and kernel methods could further improve prediction accuracy when training genomic models using multi-generation data. Using four years of doubled haploid maize data from the International Maize and Wheat Improvement Center (CIMMYT), we found that when predicting grain yield the KBLUP outperformed the GBLUP, and that using SSI with additive relationships (GSSI) lead to 5–17% increases in accuracy, relative to the GBLUP. However, differences in prediction accuracy between the KBLUP and the kernel-based SSI were smaller and not always significant.

## Introduction

Almost two decades have passed since Genomic Selection (GS) was first proposed by Meuwissen et al. (2001). This groundbreaking idea was quickly adopted for breeding dairy cattle (Hayes et al. 2009), beef cattle (Garrick 2011), broilers (Wolc et al. 2016), maize (Bernardo and Yu 2007), wheat (Poland et al. 2012), and many other animal and crop species (Xu et al. 2020). Over time, investments by public and private organizations led to the development of large genomic data sets comprising DNA sequences and phenotypes. These large sample sizes of modern genomic data sets have increased our ability to accurately train high-dimensional genomic prediction models and methods (Howard et al. 2019).

However, a larger sample size often comes with increased genetic heterogeneity, including many generations of data and often complex admixture patterns. Moreover, there have been some indications that in genomic prediction, *bigger may not always be better*. For example, Wolc et al. (2016) reported that the accuracy of genomic predictions in a broiler breeding program was higher when using data from the last three generations, relative to prediction equations trained using data from the last five generations. Likewise, Riedelsheimer et al. (2013) and Jacobson et al. (2014) reported that the prediction accuracy was higher when models were trained using data from biparental families that shared at least one parent, relative to training using data from all the available biparental families.

Early work by Habier et al. (2010) showed that family relationships have an important impact on prediction accuracy and many studies have proven that distantly related individuals make a small (sometimes negligible) contribution to the prediction accuracy. However, as noted above, some evidence suggests that using training sets formed by individuals distantly related to the genotypes of the prediction set may actually have a negative impact on the prediction accuracy (e.g., Lorenz and Smith 2015). This may happen if, for example, heterogeneity in allele frequency and in linkage disequilibrium (LD) patterns between the training and prediction sets lead to SNP-effect heterogeneity.

Issues related to data- and effect-heterogeneity have spawned multiple research efforts. One line of research models heterogeneity of effects by explicitly using SNP-by-group interaction models or multivariate models (‘multi-breed genomic prediction’), in which effects are assumed to be correlated among groups (e.g., Olson et al. 2012; Lehermeier et al. 2015; Rio et al. 2020). This approach has shown promise, yet it is only adequate when genotypes can be clustered into clearly disjointed groups.

Another line of research attempts to increase prediction accuracy with the optimal design of training data sets. The methods proposed and used to identify an optimal training set span from simple threshold-based methods (e.g., Clark et al. 2012; Lorenz and Smith 2015) to more sophisticated algorithms that seek to minimize prediction error variance or maximize the expected reliability (e.g., Rincent et al. 2012; Akdemir and Isidro-Sanchez 2019; Roth et al. 2020). A main assumption of these training set optimization methods is that a single training set is optimal for all individuals in the prediction set. However, this may not be the case if some individuals in the training set can improve prediction accuracy for some of the selection candidates and reduce it for others.

To address the limitations of existing methods, Lopez**-**Cruz and de los Campos (2021) proposed a prediction method (sparse selection index, SSI) that identifies a customized training set for each individual in the prediction set. The SSI integrates into the selection index methodology (Smith 1936; Hazel 1943) a sparsity-inducing penalty that leads to sparse selection indices and, applied to two wheat data sets, outperformed the genomic-BLUP (GBLUP; VanRaden 2008) by 5–10%.

Reproducing Kernel Hilbert Spaces (RKHS) regression has shown good predictive performance in many genomic applications (e.g., de los Campos et al. 2010; Gonz**á**lez-Camacho et al. 2012). The GBLUP is a special case of RKHS regression in which a linear kernel (additive genomic relationships) is used to describe the genetic similarity between genotypes (de los Campos et al. 2009). However, several studies (e.g., Crossa et al. 2010; de los Campos et al. 2010; Morota and Gianola 2014; Bandeira e Sousa et al. 2017; Cuevas et al. 2016, 2017, 2018) have suggested that using non-linear kernels (e.g., Gaussian kernels) may lead to higher genomic prediction accuracy. In a Gaussian kernel, the covariance between genetic values is higher for closely related individuals and drops as genotypes become increasingly distant. The rate at which the prior covariance between genetic values drops is controlled by a bandwidth parameter. Large bandwidth parameter values (that lead to highly local covariances) can be used to derive predictions that are largely dependent on closely related individuals. Thus, there is a clear link between the RKHS with Gaussian kernels and the SSI methodology of Lopez**-**Cruz and de los Campos (2021). However, the Gaussian kernel does not yield strictly sparse prediction equations.

Therefore, we study whether the SSI can also improve the prediction performance of RKHS regressions with non-linear kernels. The objective of this study is to evaluate the performance of the SSI using additive (GSSI) and non-additive (KSSI) kernels using four-generations (years) of a DH (doubled haploid) maize data set from the ongoing maize breeding program at the International Maize and Wheat Improvement Center (CIMMYT). For several scenarios of training set composition, we compare the prediction accuracy of the BLUP and the SSI with additive and non-additive kernels.

## Materials and methods

### Genotypes and phenotypic data

**Fig. 1:**
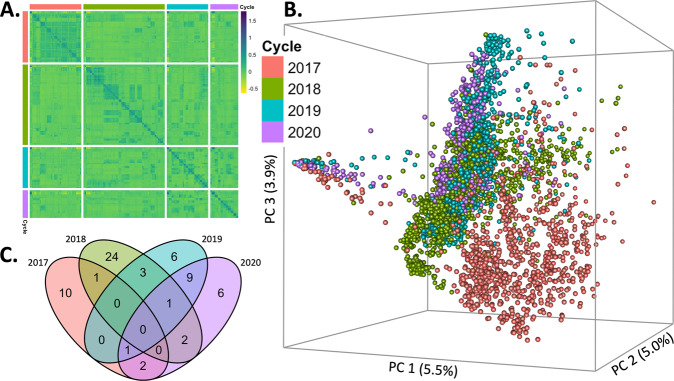
Genetic structure of the multi-generation maize data. **A** Heatmap of the genomic relationships matrix. **B** First three principal components of the additive genomic relationships matrix, **G**. Dots represent individuals that are separated by colors for each cycle (2017, 2018, 2019 or 2020). **C** Venn diagram representing the number of common parents used to generate the DH lines at each year.

The genotypes used in the study consist of 3722 DH lines derived from 54 biparental families. The DH lines were developed at CIMMYT’s Maize DH facility at the Agricultural & Livestock Research Organization (KALRO) in Kiboko, Kenya. The biparental families were obtained by crossing elite inbred lines with drought-tolerant lines that were tested for the last four years. Some parents used to generate the DH lines in one year were also used (based on a 100% pedigree similarity) to create the DH lines in other years (see Fig. 1C). There was one parent in common between 2017 and 2018; two common parents between 2017 and 2020; one common parent between 2017, 2019, and 2020; three common parents between 2018 and 2019; two common parents between 2018 and 2020; one common parent between 2018, 2019, and 2020; and finally, nine common parents between 2018 and 2019. The 3722 DH lines were selected from a larger population (based on the results of evaluating germination, good stand, plant type, low ear placement, and well-filled ears) for stage I multi-location yield trials conducted from 2017 to 2020.

Each year, the selected DH lines were crossed (as male) with a single-cross tester (as female) from the complementary heterotic group to generate tree-way hybrids that were evaluated under well-watered (denoted as *optimal*) and *drought* conditions. The number of hybrids (trials) planted in 2017, 2018, 2019, and 2020 were 923 (14), 1423 (34), 722 (17), and 654 (13), respectively; trials were connected by three to six commercial checks, planted in an alpha-lattice design with two replications and evaluated in two well-watered locations and one managed drought stress location during the 2017, 2018, 2019, and 2020 growing seasons. The optimal experiments were conducted during the rainy season, applying supplemental irrigation as needed. The drought experiments were conducted during the dry (rain-free) season, and irrigation was suspended two weeks before flowering and until harvest. Entries were planted in two-row plots, 5 m long, with a spacing of 0.75 m between rows and 0.25 m between hills. Two seeds were initially planted per hill and afterward, three weeks after emergence, one plant was kept per hill to obtain a final plant density of 53333 plants ha^−1^. Fertilizers were applied at the rate of 60 kg N and 60 kg P_2_O_5_ ha^−1^, as recommended for the area. Nitrogen was applied twice: once when planting, and again 6 weeks after emergence. Fields were kept free of weeds by hand weeding. Grain yield (GY, tons ha^−1^), anthesis date (AD, days) and plant height (PH, cm) traits were recorded. Plots were manually harvested and GY was corrected to a moisture of 12.5%. AD was measured from planting to the moment in which 50% of the plants shed pollen, and PH was measured between the soil surface and the flag leaf collar on five representative plants in each plot.

Leaf samples were taken from each of the 3722 DH lines and sent to Intertek, Sweden, for DNA extraction. The DNA sample plates were forwarded to the Institute for Genomic Diversity, Cornell University, Ithaca, NY, USA, for genotyping with repetitive sequences (rAmpSeq) as described by Buckler et al. (2016). A distortion segregation analysis was performed to a total of 5465 dominant markers coded as 0 (absence) and 1 (presence) to detect if the segregation patterns deviate from the expected Mendelian ratio of 3:1. A Benjamini–Hochberg False Discovery Rate correction was applied to the *P* values to account for multiple testing; a total of 61 markers were discarded at a 5% FDR. The remaining markers were filtered by minor allele frequency (MAF < 0.05), leading 4612 filtered markers that were used for analyses.

Data from the 2017 and 2018 cycles have been previously described and analyzed by Beyene et al. (2019) and Atanda et al. (2021).

### Phenotypes’ pre-processing

The adjusted means of GY, AD and PH were obtained using mixed-effects models fitted separately for each trait-environmental-condition-year combination. The Best Linear Unbiased Estimates (BLUE) of genotypes for the optimal experiments were estimated within year across the two locations using the META-R software (Alvarado et al. 2020) following the linear mixed model:

$$Y_{ijkl} = \mu + {\mathrm{G}}_i + {\mathrm{L}}_j + {\mathrm{R}}_{k\left( j \right)} + {\mathrm{B}}_{l\left( {kj} \right)} + \left( {{\mathrm{G}} \times {\mathrm{L}}} \right)_{ij} + e_{ijkl}$$

where *Y*_*ijkl*_ is the phenotypic record of genotype *i* in location *j* in replicate *k* within block *l*, µ is the overall mean, L_*j*_ is the fixed effect of location *j*, R_*k*(*j*)_ is the fixed effect of the replicate *k* within location *j*, B_*l*(*kj*)_ is the random effect of the incomplete block *l* within replicate *k* and location *j* assumed to be independently and identically (iid) normal distributed with a mean of zero and a variance of $\sigma _b^2$, G_*i*_ is the fixed effect of genotype *i*, (G × L)_*ij*_ is the fixed effect of the genotype × location interaction, and e_*ijkl*_ is the random error assumed to be iid normal with mean zero and variance $\sigma _e^2$. After fitting the model just described, adjusted phenotypes (*y*_*i*_) were obtained by subtracting the estimated effects of location, replicate, incomplete block, genotype × location interaction and error from each phenotypic record. Likewise, within each year, the BLUE for each trait for the single-location drought experiment was obtained through the linear model

$$Y_{ikl} = \mu + {\mathrm{G}}_i + {{{{{\mathrm{R}}}}}}_k + {{{{{\mathrm{B}}}}}}_{l\left( k \right)} + e_{ikl}$$

where R_*k*_ is the fixed effect of the replicate *k*, B_*l*(*k*)_ is the random effect of the incomplete block *l* within replicate *k* assumed to be *iid* normal with a mean of zero and a variance $\sigma _b^2$, and the remaining factors are as before. The adjusted phenotypes were obtained by subtracting the estimated effects of replicate, incomplete block, and error from the phenotypic records.

A total of *n* = 3527 lines containing marker information and phenotypic information were kept for GS models. The final number of lines in 2017, 2018, 2019, and 2020 were *n*_1_ = 901, *n*_2_ = 1418, *n*_3_ = 722, and *n*_4_ = 486, respectively.

### Genomic prediction methods

We considered four prediction models: genomic-BLUP (GBLUP) using additive genomic relationships (VanRaden 2008); Reproducing Kernel Hilbert Spaces (RKHS) regression (Gianola et al. 2006; de los Campos et al. 2010), which is equivalent to a GBLUP with a non-linear kernel (de los Campos et al. 2009); and sparse selection indices (SSI) obtained by imposing an L1-penalty on a selection index using additive genomic relationships (GSSI) and using a Gaussian kernel (KSSI). These models are described below; for simplicity, since all phenotypes were centered, we present models without intercept nor fixed effects. With the crossing and experimental design used, there were no common hybrids across years; therefore, the mixed-effects models did not include *genotype* × *year* interaction terms to account for its variability and relied only on genetic relationships among individuals.

#### GBLUP model

After adequate centering, the standard GBLUP model is represented by the following equation

1 $${\boldsymbol{y}}= {\boldsymbol{u}} + {\boldsymbol{\varepsilon}}$$

where ***y*** = [*y*_1_,…,*y*_n_]′, ***u*** = [*u*_1_,…,*u*_*n*_]′, and **ε** = [*ε*_1_,…,*ε*_*n*_]′ are t he vectors of adjusted phenotypes, breeding values (BV) and environmental error terms, respectively. Breeding values and errors are assumed to be normally distributed ${\boldsymbol{u}}\sim N\left( {{\mathbf{0}},\sigma _u^2{\mathbf{G}}} \right)$ and ${\mathbf{\varepsilon}} \sim N\left( {\mathbf{0},\sigma _\varepsilon ^2{{{{{\mathbf{I}}}}}}} \right)$, where $\sigma _u^2$ and $\sigma _\varepsilon ^2$ are the genetic and error variances, respectively, **G** is the additive genetic relationship matrix (GRM), and **I** is an identity matrix.

The genomic relationship matrix **G** = {*g*_*ij*_} was derived from markers, **X** = {*x*_*im*_} using **G** = **ZZ**′/*p*, where *p* = 4673 is the number of markers and ${{{{{\mathbf{Z}}}}}} = \left\{ {\left( {x_{im} - \overline x _m} \right)/sd_{x_m}} \right\}$ is the matrix of centered and scaled markers obtained by subtracting the mean of the corresponding column from each marker entry, followed by scaling by the standard deviation of the column.

The predicted BVs ($\widehat {\boldsymbol{u}}_{PS}$) for the individuals in the prediction set (PS) are then linear combinations of the phenotypes of the cultivars in the training set (TS, ***y***_*TS*_) (Searle et al. 1992) such that

2 $$\widehat{ \boldsymbol{u}}_{PS} = {\mathbf{B}}_G{\boldsymbol{y}}_{TS}$$

where ${{{{{\mathbf{B}}}}}}_G = {{{{{\mathbf{G}}}}}}_{PS,TS}\left( {{{{{{\mathbf{G}}}}}}_{TS} + \lambda _0{{{{{\mathbf{I}}}}}}} \right)^{ - 1}$ is a Hat matrix (i.e., coefficients of regression of BVs on phenotypes), $\lambda _0 = \sigma _\varepsilon ^2/\sigma _u^2$ is the ratio between residual and genetic variances, ${{{{{\mathbf{G}}}}}}_{PS,TS}$ is a matrix containing the additive genetic relationships between the data points in the prediction set and those in the training set, and **G**_*TS*_ represents the additive GRM of the training data points. The predictions of the GBLUP (given by Eq. (2)) can be shown to be equivalent to those of a selection index (e.g., Henderson 1977; Lopez**-**Cruz and de los Campos 2021).

#### RKHS models

RKHS regression is equivalent to the GBLUP model just described, with **G** replaced by any positive-definite kernel (**K**). Here, we used a Gaussian kernel **K** = {*K*_*ij*_(*θ*)} (*i*,*j* = 1,…, *n*) where $K_{ij}\left( \theta \right) = {{{{{\mathrm{exp}}}}}}\left( { - \theta \widetilde d_{ij}^2} \right)$. Here *θ* is a bandwidth parameter and $\widetilde d_{ij}^2$ is the scaled squared Euclidean distance between individuals *i* and *j* given by their marker genotypes, obtained by dividing the distance $d_{ij}^2 = \mathop {\sum}\nolimits_{m = 1}^p {\left( {x_{im} - x_{jm}} \right)^2}$ by the average distance $\overline d = \frac{1}{{n^2}}\mathop {\sum}\nolimits_i {\mathop {\sum}\nolimits_j {d_{ij}^2} }$. Following Gonz**á**lez-Camacho et al. (2012) we generated three different kernels **K**_1_ = {*K*_*ij*_(*θ*_1_)}, **K**_2_ = {*K*_*ij*_(*θ*_2_)}, and **K**_3_ = {*K*_*ij*_(*θ*_3_)}, where *θ*_1_ = 0.2, *θ*_2_ = 1, and *θ*_3_ = 5.

In addition to the single-kernel models above-described, we also considered a multi-kernel model (aka, ‘kernel averaging’, KA; de los Campos et al. 2010) with the three kernels previously described. Briefly, The KA model includes three random effects, ***y*** = ***u***_1_ + ***u***_2_ + ***u***_3_ + ***ε***, each following a normal distribution with its own variance parameter, ${{{{{\boldsymbol{u}}}}}}_k\sim N\left( {{\mathbf{0}},\sigma _{a_k}^2{{{{{\mathbf{K}}}}}}_k} \right)$ (*k* = 1,2,3).

#### Sparse selection index

This approach was recently introduced by Lopez**-**Cruz and de los Campos (2021). The methodology introduces an L1-sparsity-inducing penalty into a selection index problem. Here, in an SSI using additive relationships (GSSI), the weights of the selection index for the *i*^*th*^ individual in the prediction set was obtained from the following L1-penalized optimization problem

3 $$\widetilde {{{{{\boldsymbol{b}}}}}}_{i_G}\left( \lambda \right) = \mathop {{{{{{{\mathrm{arg}}}}}}\,{{{{{\mathrm{min}}}}}}}}\limits_{{{{{{\boldsymbol{b}}}}}}_i} \left\{ {\frac{1}{2}{{{{{\boldsymbol{b}}}}}}_i^\prime \left( {\mathbf{G}}_{TS} + \lambda _0{\mathbf{I}} \right){\boldsymbol{b}}_i - {\mathbf{G}}_i^\prime {\boldsymbol{b}}_i + \lambda \mathop {\sum}\nolimits_{j = 1}^n {\left| {b_{ij}} \right|} } \right\}$$

where ${{{{{\mathbf{G}}}}}}_i^\prime = {{{{{\mathbf{G}}}}}}_{PS\left( i \right),TS}$ is the vector containing the additive relationships between the *i*th subject in the prediction set and each of the subjects in the training set, *λ* is a parameter controlling the degree of sparsity of $\widetilde {{{{{\boldsymbol{b}}}}}}_i$, and $\mathop {\sum}\nolimits_{j = 1}^n {\left| {b_{ij}} \right|}$ is an L1-penalty on the coefficients **b**_*i*_. A (sparse) Hat matrix for the GSSI, $\widetilde {\mathbf{B}}_G\left( \lambda \right)$ contains, in each row, the solutions to Eq. (3), obtained for each testing line, i.e., $\widetilde {\mathbf{B}}_G\left( \lambda \right) = \{ {\widetilde {{\boldsymbol{b}}}_{i_G}\left( \lambda \right)^\prime }\}$. A value of *λ* = 0 yields the same (non-sparse) Hat matrix as the standard GBLUP in Eq. (2).

For the SSI with a Gaussian kernel (KSSI), we used Eq. (3) with the kernel (either **K**_1_, **K**_2_, **K**_3_, or **K**_A_) instead of the additive relationship matrix (**G**) to obtain a sparse Hat matrix $\widetilde {\mathbf{B}}_K\left( \lambda \right) = \{ {\widetilde {{\boldsymbol{b}}}_{i_K}\left( \lambda \right)^\prime }\}$.

Although the optimization problem in Eq. (3) does not have a closed form, solutions can be derived using a coordinate descent algorithm (Lopez**-**Cruz et al. 2020). Finally, an optimal value of *λ* can be obtained using cross-validation within the training set.

### Variance components

The implementation of GBLUP, KBLUP and the corresponding SSIs requires estimates of variance components. These estimates were obtained by fitting Bayesian genomic models into each trait-environment combination. These analyses were performed using the BGLR R-package (Perez and de los Campos 2014) with the default setting for hyper-parameters. After fitting the models, posterior means of the variance components were obtained. For the standard KABLUP, the model was fitted with the three kernels together to estimate the kernel-specific variances and then used to derive $\sigma _a^2 = \sigma _{a_1}^2 + \sigma _{a_2}^2 + \sigma _{a_3}^2$ and the average kernel ${{{{{\mathbf{K}}}}}}_A = \frac{{\sigma _{a_1}^2}}{{\sigma _a^2}}{{{{{\mathbf{K}}}}}}_1 + \frac{{\sigma _{a_2}^2}}{{\sigma _a^2}}{{{{{\mathbf{K}}}}}}_2 + \frac{{\sigma _{a_3}^2}}{{\sigma _a^2}}{{{{{\mathbf{K}}}}}}_3$. The proportion of the trait variance that is explained by the BLUP models was calculated as $h^2 = \sigma _u^2/\left( {\sigma _u^2 + \sigma _\varepsilon ^2} \right)$ where $\sigma _u^2$ can be either the additive or non-additive (kernel) genetic variance estimate. All these models were fitted using only data from the training set.

### Assessment of prediction accuracy

Variance components estimates and the corresponding GRM (**G**, **K**_1_, **K**_2_, **K**_3_, or **K**_A_) were used to derive the non-sparse (**B**_*G*_ or **B**_*K*_ for the standard BLUP) and the sparse ($\widetilde {{{{{\mathbf{B}}}}}}_G\left( \lambda \right)$ or $\widetilde {{{{{\mathbf{B}}}}}}_K\left( \lambda \right)$ for the SSI) Hat matrices. The predictions ($\widehat {{{{{\boldsymbol{u}}}}}}_{PS}$) were derived (as in Eq. (2)) as the product of the (non-sparse or sparse) Hat matrix times the vector of phenotypes in the training set. Prediction accuracy was measured as the correlation between observed and predicted values in the prediction set, i.e., $\rho = cor\left( {{{{{{\boldsymbol{y}}}}}}_{PS},\widehat {{{{{\boldsymbol{u}}}}}}_{PS}} \right)$.

The prediction accuracy was assessed for different prediction scenarios using cycle 2020 as the prediction set with different training set compositions using data from previous generations, as follows: (*i*) the data from the 2020 cycle was randomly partitioned into 85–15% (i.e., 413 and 73 individuals), (*ii*) the 85%-set (*n*_*PS*_ = 413) from the year 2020 was predicted using data of the single year 2017 (*n*_*TS*_ = 901), 2018 (*n*_*TS*_ = 1418), or 2019 (*n*_*TS*_ = 722), or cumulated years 2018+2019 (*n*_*TS*_ = 2140) or 2017+2018+2019 (*n*_*TS*_ = 3041) as a training set, (*iii*) the prediction of the 413 individuals was also performed using the same training sets, but augmented by progressively including the remaining 15%-set from 2020, first 25 (5%), then 49 (10%), and finally, 73 (15%) individuals. See Table 1 for a summary of all the training set compositions. All predictions were performed 100 times using different random partitions of the 2020 data.

**Table 1:** Training set (TS) composition used in each prediction scenario. (The prediction set was the same for all training scenarios and consisted of 413 (i.e., 85%) randomly chosen individuals from the 2020 cycle).

	% of 2020 data used for training (n)			
	0 (0)	5 (25)	10 (49)	15 (73)
TS cycle(s)	Total training size (*n*_*TS*_)			
2017	901	926	950	974
2018	1418	1443	1467	1491
2019	722	747	771	795
2018+2019	2140	2165	2189	2213
2017+2018+2019	3041	3066	3090	3114

### Software

All the aforementioned analyses were performed in the R environment-language (R Core Team 2019). All standard Bayesian GBLUP and KBLUP models were fitted using the BGLR R-package (Perez and de los Campos 2014) to estimate variance components. The sparse Hat matrices ($\widetilde {{{{{\mathbf{B}}}}}}_G\left( \lambda \right)$ or ${\widetilde{\mathbf{B}}}_K\left( \lambda \right)$) were obtained using the SFSI R-package (Lopez**-**Cruz et al. 2020). For each trait-environment partition, an optimal value of *λ* was obtained using 10-fold cross-validation within the training set.

## Results

The germplasm used in this study is derived from different biparental families across 4 years. This richness of the data is reflected in a high population heterogeneity, in which individuals cluster into groups within and across generations (Fig. 1). However, the crosses performed prevented the formation of a clear structure (e.g., 2 clusters); instead, the population shows a more cryptic substructure with varying degrees of admixture between families and generations. The intermixing between generations observed in Fig. 1 can be attributed to the connection among years through common parents leading to the formation of a varied number of half-sib families among all years.

### Prediction accuracy comparison of GBLUP and KBLUP models

**Fig. 2:**
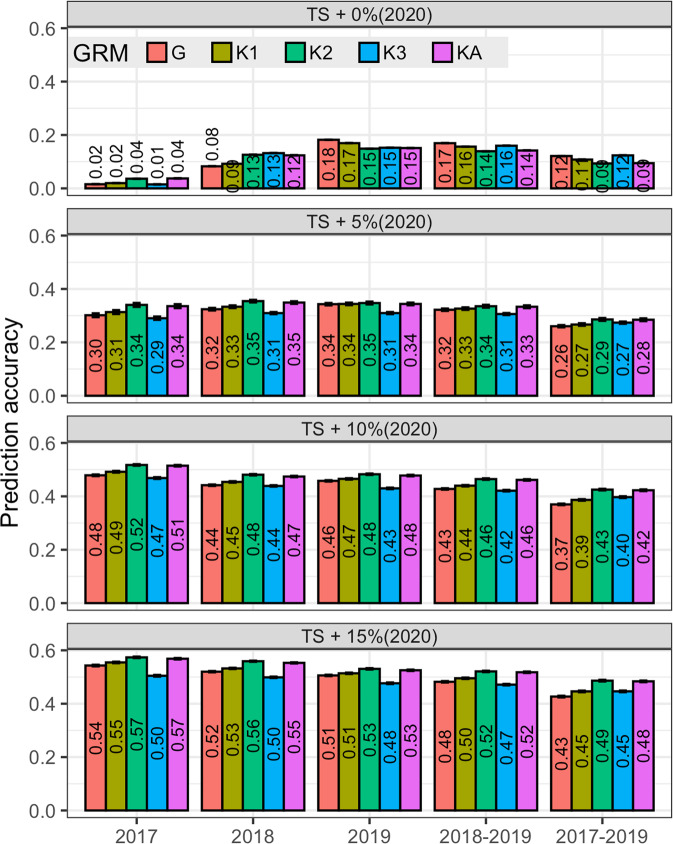
Prediction accuracy of the BLUP models by training set (TS). Models were fitted using different genetic relationship matrices (**G**, **K**_1_, **K**_2_, **K**_3_, or **K**_A_). TSs consisted of all the data from the single cycles 2019, 2018, or 2017 alone (top-left panel), or in combination with a proportion (5% = 25, 10% = 49, 15% = 73) of the data from the 2020 cycle. The prediction set consisted of 413 genotypes (representing the 85%) from the 2020 cycle that were not used for model training. Trait GY, optimal environment.

Figure 2 shows the accuracy of prediction (averaged across all 100 partitions) for GY in the optimal environment using all standard BLUP models for all different training set compositions representing a combination of previous cycles (2017, 2018, 2019, 2018–2019, or 2017–2019) plus the inclusion of 0, 5, 10, and 15% (i.e., 0, 25, 49, and 73 subjects) of the total number of individuals from the same prediction cycle 2020 (see Table 1). The lowest accuracies were observed when the training set was not referenced to the target prediction set (i.e., when no data from 2020 is included in the training set, compare the top panel with other panels in Fig. 2). As expected, the inclusion of individuals from the same prediction cycle increases the prediction accuracy across all models and training set composition. For instance, when augmenting the training set to include 25 genotypes (i.e., 5%) from 2020, the accuracy of the GBLUP increased by 88% when predicting with 2019 (0.18 vs 0.34) and by more than 100% when using 2018 (0.08 vs 0.32) and 2017 (0.02 vs 0.30) as a training set (compare the 2 top panels in Fig. 2). Furthermore, the increase in accuracy of the GBLUP is even larger (at least 180%) when adding 73 genotypes (i.e., 15%) from the 2020 cycle. The same patterns were obtained for GY-drought (Supplementary Fig. S1) in which the accuracy of the models is very low when no reference data from 2020 is included in the training set and it increases as reference data is added to the training set.

Higher prediction accuracies are achieved when using only the most recent previous generation (cycle 2019) as a training set (see the top panel in Fig. 2); this was expected as the number of parents shared between the prediction cycle 2020 and other cycles (Fig. 1C) was larger for cycle 2019 (11 parents) than with previous cycles (3 parents shared between cycle 2020 and either cycle 2018 or 2017). However, older generations (2018 or 2017), when combined with data from 2020, provided similar or even better predictions. For instance, the BLUP models trained with 2017 (plus 15% of the 2020 cycle) data yielded accuracies that are 0.02–0.04 points (in the correlation scale) higher than that of models trained with 2019 plus 15% of the 2020 data (see the bottom panel in Fig. 2).

The results when cumulated previous years (2018+2019 and 2017+2018+2019) were used as a training set to predict 2020 are also shown in Fig. 2. The highest prediction accuracies were obtained when using one generation back (i.e., cycle 2019 alone) as a training set. The inclusion of older generations in the training set did not increase but rather reduced the accuracy, compared with using only one generation back (Fig. 2). For instance, using 2018–2019 as a training set, the accuracy of the GBLUP model showed a reduction of 5–6% (0.01–0.03 points) relative to when using 2019 alone. Using three generations back (2017–2019) for model training yielded a decrease of 15–30% (0.05–0.08 points) in the accuracy. The same patterns were also observed for GY-drought (Figure S1) in which the accuracy of the models was reduced by 12–60% (0.04–0.17 points, relative to when using 2019 alone as a training set) as more older generations were cumulated in the training set.

In general, kernel-based models (especially using the Gaussian kernels **K**_1_, **K**_2_, and **K**_A_) achieved higher prediction accuracy than the standard GBLUP with increases in accuracy of 1–15% (0.01–0.06 points). Although kernels **K**_1_, **K**_2_, and **K**_3_ are ranked differently across training set compositions, models with **K**_A_ seem to be more stable across all scenarios, with a performance similar to that of the best of the three kernels **K**_1_, **K**_2_, or **K**_3_. These results are in agreement with the findings of de los Campos et al. (2010) who highlighted the importance of combining different kernels as a way to make the model robust with respect to the choice of kernel.

### Effect of sparsity on prediction accuracy

The same partitions of training-prediction sets used to obtain the results for the (non-sparse) BLUP models were used to evaluate the prediction accuracy of SSIs (sparse models). A cross-validated value λ_*CV*_ was found within the training set to calculate an optimal SSI. Table 2 contains the results of the predictions of GY-optimal trait-environment combination for the scenario in which 15% of the data from 2020 is included in the training set (either 2017, 2018, 2019, 2018–2019, or 2017–2019). Results for the cases when adding 0, 5, and 10% of the 2020 data are presented in Supplementary Tables S1–S3. With this training set composition, the accuracy of the standard GBLUP models was between 0.43 and 0.54.

**Table 2:** Accuracy of prediction for each training set (TS) composition (including 15% = 73 subjects from the 2020 cycle), trait GY, optimal environment.

TS/*n*_*TS*_	GRM	*λ*_*CV*_^a^	*n*_*sup*_^b^ (%sparsity)	*h*^2^	Accuracy (SD)		Gain 1 (%)^c^	Gain 2 (%)^d^
					BLUP	SSI		
2017 *n*_*TS*_ = 974	G	0.0175	151 (16)	0.53	0.54 (0.079)	0.56 (0.079)	–	3
	K1	0.0037	180 (18)	0.87	0.55 (0.077)	0.54 (0.089)	2	−3
	K2	0.0048	238 (24)	0.76	0.57 (0.071)	0.57 (0.072)	6	0
	KA	0.0030	293 (30)	0.85	0.57 (0.074)	0.57 (0.077)	5	0
2018 *n*_*TS*_ = 1491	G	0.0089	358 (24)	0.61	0.52 (0.064)	0.57 (0.052)	–	10
	K1	0.0017	414 (28)	0.91	0.53 (0.063)	0.57 (0.053)	2	8
	K2	0.0028	508 (34)	0.80	0.56 (0.057)	0.58 (0.053)	8	4
	KA	0.0018	498 (33)	0.88	0.55 (0.058)	0.58 (0.053)	6	4
2019 *n*_*TS*_ = 795	G	0.0357	71 (9)	0.54	0.51 (0.068)	0.54 (0.066)	–	7
	K1	0.0139	90 (11)	0.88	0.51 (0.068)	0.55 (0.066)	2	7
	K2	0.0058	207 (26)	0.73	0.53 (0.069)	0.54 (0.068)	5	2
	KA	0.0041	219 (28)	0.80	0.53 (0.069)	0.54 (0.070)	4	2
2018–2019 *n*_*TS*_ = 2213	G	0.0148	246 (11)	0.57	0.48 (0.071)	0.54 (0.063)	–	12
	K1	0.0024	373 (17)	0.90	0.50 (0.071)	0.54 (0.064)	3	8
	K2	0.0023	783 (35)	0.77	0.52 (0.070)	0.54 (0.066)	8	3
	KA	0.0016	864 (39)	0.82	0.52 (0.071)	0.54 (0.066)	7	3
2017–2019 *n*_*TS*_ = 3114	G	0.0141	322 (10)	0.52	0.43 (0.079)	0.49 (0.081)	–	14
	K1	0.0026	435 (14)	0.88	0.45 (0.081)	0.51 (0.072)	5	13
	K2	0.0023	978 (31)	0.75	0.49 (0.081)	0.51 (0.079)	14	4
	KA	0.0018	1079 (35)	0.81	0.48 (0.081)	0.50 (0.078)	13	4

*GRM* genetic relationship matrix, *SD* standard deviation, *h*^2^ proportion of the trait variance explained by the model.

^a^Penalization parameter in Eq. (3) found by cross-validating the TS.

^b^*n*_*sup*_ = average number of individuals from the TS with a non-zero coefficient in the sparse Hat matrix (support set). %sparsity = 100 × *n*_*TS*_/*n*_*sup*_. In the BLUP models, *λ*_*CV*_ is equal to zero and *n*_*sup*_ is equal to the total TS size. Within each TS cycle, percentage of increase in accuracy of ^c^the standard KBLUP relative to the standard GBLUP ($= 100 \times \frac{{KBLUP\,accuracy - GBLUP\,accuracy}}{{GBLUP\,accuracy}}$), and of ^d^the *SSI relative to the standard *BLUP ($= 100 \times \frac{{ \ast SSI\,accuracy - \ast BLUP\,accuracy}}{{ \ast BLUP\,accuracy}}$, * = G, K_1_, K_2_, or K_A_).

Standard KBLUP (with kernels **K**_1_, **K**_2_, or **K**_A_) models achieved higher prediction accuracy than the standard GBLUP, with gains in accuracy (relative to the GBLUP) ranging from minimal (0.01 points) to substantial (0.06 points). Sparse models (GSSI and KSSI) yielded even higher accuracies than the standard GBLUP, with gains in accuracy (relative to the GBLUP) ranging from 0.02 to 0.08 points (Table 2). The gains in prediction accuracy of the KBLUP models are smaller when the accuracy of the standard GBLUP models was very low. For instance, when the accuracy of the GBLUP is as low as 0.02–0.18 (the case where no data from 2020 are included in the training set, Supplementary Table S1), standard KBLUP models performed similarly or worse (0.01–0.03 reduction in accuracy) than the standard GBLUP models; however, SSIs yielded gains in accuracy (relative to the standard GBLUP) of 0.01–0.08 (Supplementary Table S1). It was only in these low-accuracy situations that, in some cases, the use of a standard KBLUP model resulted in ~0.03 loss of accuracy (relative to the standard GBLUP), and that using sparse models, the accuracy lost was 0.01–0.05 (see Supplementary Table S1).

In the situations where data from 2020 was used for model training, the sparse models (GSSI or KSSI) provided always an increase in the accuracy, relative to their corresponding non-sparse (GBLUP or KBLUP) models, of 0.02–0.08 points (Table 2 and Supplementary Tables S2 and S3); this was true only for models using additive relationships **G** and kernels **K**_1_, **K**_2_, or **K**_A_. However, when no data from 2020 is included in the training set (i.e., the low-accuracy cases), the SSIs showed a reduction (relative to the standard BLUPs) in the accuracy of 0.03–0.05 points (Supplementary Table S1). No significant difference between sparse and non-sparse models was observed when using the large-bandwidth kernel **K**_3_.

**Fig. 3:**
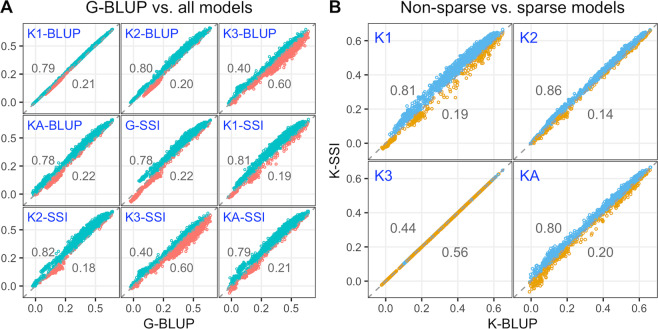
Point-wise prediction accuracy comparison between models. **A** Prediction accuracy of the standard (non-sparse) GBLUP model (horizontal axis) versus the prediction accuracy of all other models (vertical axis of each panel). **B** Prediction accuracy of the standard KBLUP model (horizontal axis) versus the prediction accuracy of the KSSI (vertical axis) by type of kernel used in panels. Each point represents a training-testing partition within each training set composition. Colored dots (numbers) above (below) the 45-degree line represent cases (the proportion) for which one model outperformed the other model. Trait GY, optimal environment.

In summary, across all scenarios, the standard GBLUP showed the lowest accuracy among all models (except the **K**_3_-based models, see Fig. 3A). This inferiority of the standard GBLUP was also observed for GY in the drought environment (see Supplementary Fig. S2A). The addition of sparsity to the KBLUP models resulted sometimes (in at least 80% of the cases) in an increased accuracy when the kernel used a small bandwidth (**K**_1_ with *θ* = 0.2, and **K**_2_ with *θ* = 1) or when averaged across extreme kernels (**K**_A_) for optimal and drought environments (Fig. 3B and Supplementary Fig. S2B).

### Automatic training-sample selection

Table 2 (and Supplementary Tables S1–S3) show the optimal value of the penalization parameter *λ* and the degree of sparsity of the resulting SSI, measured by the average number of subjects from the training set in the support set (*n*_*sup*_, subjects with a non-zero coefficient in the estimated Hat matrix) of each predicted genotype. The degree of sparsity varied across models. For GY in optimal environments, across all training set compositions, the strongest sparsity was achieved using the genomic matrix **G** with a relative sparsity (*n*_*sup*_/*n*_*TS*_) of 4–42% (Table 2 and Supplementary Tables S1–S3), while the relative sparsity of the KSSIs increases as the bandwidth parameter *θ* increases (relative sparsity of 9–52% for **K**_1_ and 20–62% for **K**_2_). The relative sparsity achieved when using the **K**_A_ kernel (21–59%) was similar to that of the **K**_2_ kernel (see Table 2 and Supplementary Tables S1–S3). The fact that no differences in accuracy were observed between the standard K_3_BLUP and K_3_SSI is due to the fact that the optimal *λ*_*CV*_ was zero; thus, the sparse model was equivalent to the standard model.

**Fig. 4:**
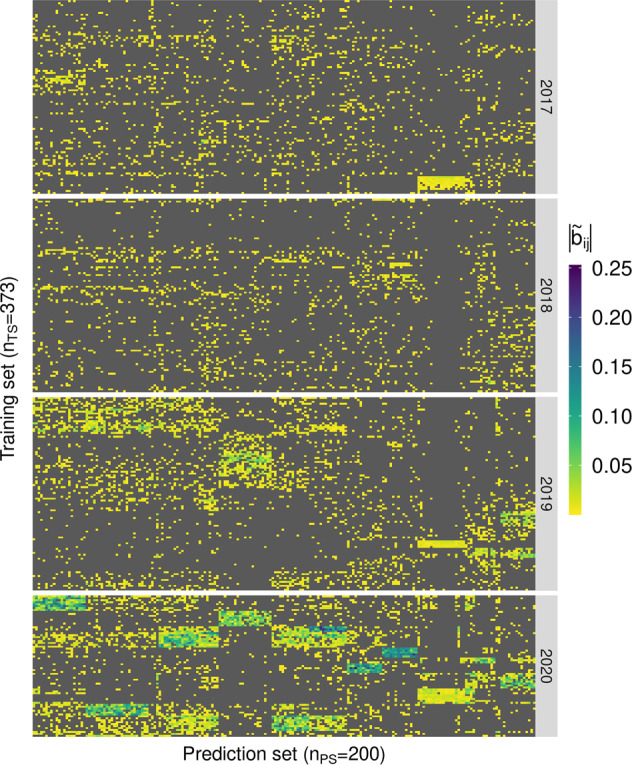
Heatmap of the coefficients in the Hat matrix ($\widetilde {{\mathbf{B}}}_G\left( \lambda \right)$) of the GSSI for one training-prediction (TS-PS) partition in the prediction of *n*_*PS*_ = 413 individuals from 2020 using *n*_*TS*_ = 3114 individuals (2017+2018+2019 plus 15% = 73 genotypes from the 2020 cycle). Columns represent (a sample of 200) predicted individuals and rows represent (a sample of 100 individuals from each cycle 2017–2019 and the 73 subjects from 2020) training individuals, separated by cycle. Each column vector represents values of the vector $\widetilde {{\boldsymbol{b}}}_{i_G}\left( \lambda \right) = \{ {\widetilde b_{ij}}\}$, *j* = 1,…,3114 (Eq. (3)) using a value of *λ* obtained by cross-validation. Individuals not contributing to the prediction have a coefficient $\widetilde b_{ij} = 0$ and are in gray. Individuals with a non-zero coefficient are shown in a yellow-blue scale. Trait GY, optimal environment.

Figure 4 displays a heatmap of the sparse Hat matrix ($\widetilde {{\mathbf{B}}}_G\left( \lambda \right)$) of the GSSI. Selected individuals in the training set (2017–2019 plus 15% of data from 2020) appear in rows and those in the prediction set are shown in columns. Individuals from the training set that did not contribute to each SSI (i.e., those with zero weight in the index) are displayed in gray. Those with a non-zero coefficient (support set) are shown in a yellow-blue scale. The heatmap shows how SSIs select custom training sets for each genotype in the prediction set. Individual genotypes in the training set support the prediction of some, though not all the genotypes in the prediction set. The solution for the Hat matrix in Fig. 4 is very sparse, with a varying number of support points (comprising data from all generations) by testing genotype. Training genotypes from the same prediction cycle 2020 are more important for prediction (as measured by the magnitude of the regression coefficient) as they are more closely related to testing individuals. These individuals had the higher coefficients in the Hat matrix of the GBLUP and were not zeroed-out in the GSSI (see Supplementary Fig. S3). Prediction of each of the 413 testing genotypes was performed using phenotypes from an average of 322 (out of 3114, i.e., 10%; see Table 2) training genotypes. For the same prediction scenario, a heatmap for the KSSI with kernel **K**_*A*_ (showing 35% sparsity) is provided in Supplementary Fig. S4.

**Fig. 5:**
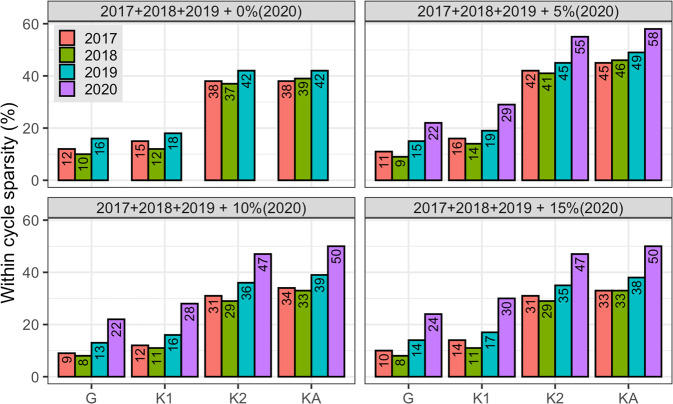
Proportion of the training individuals from each cycle that contributed to the prediction of genotypes from 2020 (averaged across all the 413 testing subjects), using SSIs with different relationship matrices (**G**, **K**_1_, **K**_2_, or **K**_*A*_). The training set was composed of individuals from 2017 (*n* = 901), 2018 (*n* = 1418), and 2019 (*n* = 722) alone (top-left panel), or in combination with a proportion (5% = 25, 10% = 49, 15% = 73) of the data from the 2020 cycle. Trait GY, optimal environment.

Figure 5 shows, for each of the sparse models, the proportion of the training individuals from each cycle (2017, 2018, or 2019) that contributed to the prediction (within the cycle support set) of the testing individuals. Each panel represents the different training sets composed of 2017+2018+2019 data plus the addition of either 0, 5, 10, or 15% of the 2020 data. As expected, training individuals that belong to the same group as the testing individuals are more likely to be included in the support set. For example, using a GSSI trained with 2017–2019 plus 5% from the 2020 data (see the top-right panel in Fig. 5), on average, 22% of the 25 genotypes from 2020 included in the training set contributed to the prediction of the testing individuals. Although more abundant, a smaller portion of the total individuals from previous cycles (11% of the 901 subjects from 2017 cycle, 9% of the 1418 from 2018, and 15% of the 722 from 2019) also contributed to the prediction. With a smaller degree of sparsity, similar patterns were also observed for the KSSIs (Fig. 5) except with **K**_3_, which rendered no sparsity (not shown in the figure). Plots displaying the within-cycle sparsity patterns for GY in the drought environment are shown in Supplementary Fig. S5.

As more individuals from the same prediction cycle are added to the training set, fewer individuals from previous generations become less frequent in the support set. For instance, performing the prediction with a GSSI using 2017–2019 cycles including 15% of the 2020 data (bottom-right panel in Fig. 5), yielded a smaller support set with only 10% (90 of 901) of the 2017 data, 8% (114 of 1418) of the 2018 data, and 14% (101 of 722) of the data from 2019; however, data from 2020 became more frequent in the support set with 24% (18 of 73 subjects). This happened because the SSI zeroed-out the regression coefficient (b_*ij*_) of training individuals that, in the Hat matrix of the BLUP (Eq. (2)), are already relatively small in absolute value and that have a small absolute genomic relationship (*g*_*ij*_) with individuals in the prediction set (i.e., training individuals in a neighborhood of the origin); these coefficients correspond mostly to individuals from previous cycles (see Supplementary Fig. S3).

## Discussion

Multiple factors can affect the predictive performance of GS models, including sample size, trait heritability, the extent of LD between markers and quantitative trait loci (QTL), as well as the relationships between training and testing genotypes (Daetwyler et al. 2008; Heffner et al. 2009; Lorenzana and Bernardo 2009; Combs and Bernardo 2013).

General guidelines suggest that prediction accuracy is maximized when the training set includes a sufficiently large number of individuals distantly related to each other (Rincent et al. 2012) and closely related to the subjects in the prediction set (Habier et al. 2010; Clark et al. 2012). On the other hand, there is evidence suggesting that increasing the training set size by including individuals that are genetically distant to those in the prediction set does not necessarily increase, and might even reduce, the prediction accuracy (e.g., Lorenz and Smith 2015).

Each cycle of a breeding program produces a new batch of genotype/phenotype data; therefore, after many years of adopting GS, the data available for model training are typically multi-generational and may often include complex patterns of pedigree relationships within and between generations. There is clear evidence that a GS model needs to be re-trained every cycle (Wolc et al. 2011; Wientjes et al. 2013; Pszczola and Calus 2016). When re-training models, breeding organizations face many challenges. Should all the available data be used for model training? Should researchers restrict the training data to only include genotypes/phenotypes from recent generations? Or should they exclude data from genotypes distantly related to the current set of selection candidates?

Some evidence suggests that in genomic prediction, ‘bigger is not necessarily better’. For instance, using historic wheat data generated over 17 years, Dawson et al. (2013) observed that the accuracy of year-to-year predictions using training sets composed of all previous years was approximately the same as when considering only three years back. Likewise, in a broiler breeding population, Wolc et al. (2016) found that the maximum accuracy was accomplished when the training set was composed of the three most recent generations.

The SSI methodology offers a framework to identify a customized training set (or support points) for each individual in the prediction set, from which the predictions are derived. This methodology considers both the relationships between the candidate for selection and each training genotype, as well as relationships between training genotypes (Eq. (3)). Therefore, we suggest that the SSI can be used to address the problem of training set optimization with multi-generation data. In this research we used multi-generation data originated from more than 50 biparental families to measure the impact of sparsity using SSIs formed using additive and non-additive kernels. We corroborated that the use of all available multi-generation information together can decrease the prediction accuracy of the standard GBLUP as the data become increasingly heterogeneous (see Fig. 2 and Supplementary Fig. S1) where the performance of genotypes is also affected by the usual variability in weather conditions from one year to another (not applicable in this study).

The accuracy of markers-derived predictions is provided by family relationships and by the LD between QTL and markers. These two sources of accuracy are difficult to separate (Habier et al. 2007; Habier et al. 2010). Increasing marker density can increase the proportion of the trait variance explained by BLUP models; however, closing the gap between the trait- and SNP-heritability requires having markers in high LD with causal variants (Makowsky et al. 2011; Kim et al. 2017). We observed that, when augmenting the training set by adding only a few individuals from the same cycle, the accuracy increased but the proportion of variance explained by the model remained unchanged (compare Table 2 and Supplementary Tables S1–S3); suggesting a sizable contribution of closely related individuals (i.e., individuals from the same cycle) to prediction accuracy (see Supplementary Fig. S3). These results are in agreement with Habier et al. (2007) who found that the accuracy of BLUP models is mostly resulted from the genetic relationships between training and testing individuals, with a bare contribution from LD.

One could expect that models that have higher proportion of variance explained in the training set (estimated using a ratio of variance components) may also achieve a higher prediction accuracy. However, this is not necessarily the case as there may be over-fitting or because prediction accuracy also depends on the accuracy of estimated effects (e.g., Goddard 2009; Makowsky et al. 2011). In general, the RKHS models fitted the training data better (these models had 0.20–0.35 points higher ‘heritability’ than the GBLUP, see Table 2). However, the difference in prediction accuracy between the linear and non-linear models was more modest; thus, suggesting that RKHS models either over-fitted the data or were more complex and, therefore, with the same training set, achieved a lower accuracy of estimates of effects.

Our results confirmed that an SSI based on additive relationships yields a higher prediction accuracy than the standard additive GBLUP. When a non-additive kernel was used, we found that sparsity improved prediction accuracy, provided that the kernel used was not already a ‘local’ kernel, that is, a kernel in which genetic covariances are positive only for closely related individuals. For local kernels (**K**_3_), adding sparsity did not improve prediction accuracy in a clear and systematic manner. Therefore, our results confirm the benefit of ‘local predictions’ (largely dependent on closely related individuals), which can be obtained either by using an RKHS with local kernels or with an SSI applied to additive genomic relationships.

Both the SSI and the Kernels regression require the optimization of a parameter that controls local predictions. The SSI requires the optimization of the penalization parameter (λ), which can be done by cross-validation within the training data set. On the other hand, the RKHS regression requires the bandwidth parameter, which controls how fast covariances drop with genetic distance, to be tuned. This can be done either by comparing multiple kernels using cross-validation or by using multiple kernels with ‘kernel averaging,’ as discussed in de los Campos et al. (2010).

Standard training-optimization methods assume that a single training set is optimal for all selection candidates. The SSI does not make this assumption. Our results clearly show that each SSI picks a particular set of support points and that the optimal training set varies between lines in the prediction set. The inspection of the Hat matrix of the SSI (see Fig. 4 and Supplementary Fig. S4) makes it clear that in prediction, *one-size-does-not-fit-all* selection candidates. Likewise, the inspection of the Hat matrix shows that optimizing training sets by restricting the training data to recent generations may also not be optimal. Indeed, most of the SSIs picked information from all the generations available, with varying levels of sparsity. Lopez**-**Cruz and de los Campos (2021) found that the SSI provided higher gains in accuracy (over the GBLUP) as the number of training subjects increased (keeping marker density and population structure fixed). Their results demonstrated that the SSI can be more advantageous over BLUP models in situations when data exhibits a high degree of heterogeneity and/or is very large. Under these conditions, the SSI has more opportunities to detect an optimal training set providing increased accuracy.

### Computational considerations

For a given training and testing set, computing an SSI for *n*_*PS*_ testing genotypes involves solving a penalized problem (in Eq. (3)) *n*_*PS*_ times. This is generally computationally more costly than solving BLUP equations (Eq. (2)) for the same training and testing sets. However, it is worth noting that the task of solving SSIs for *n*_*PS*_ genotypes can be fully parallelized in *n*_*PS*_ independent tasks. The computational cost of solving the SSI for one testing genotype depends on the training size and on the optimal value of the regularization parameter λ (highly sparse indices are very fast to compute). In a Linux-based system with a 2.4 GHz Intel Xeon Gold processor and 32 GB of RAM, solving the penalized problem for a single testing individual using all three previous years plus 15% of 2020 data (*n*_*TS*_ = 3114) as training took on average 0.11 s for the GSSI and 2.01 s for the K_A_SSI (see Supplementary Fig. S6). As noted, all these computations can be fully parallelized in a high-performance computing environment. For instance, calculating a GSSI for all the *n*_*PS*_ = 413 testing individuals can take around $413 \times \frac{{0.11}}{{10}} = 4.5$ seconds with parallel computing using 10 cores, while it can take up to $413 \times \frac{{2.01}}{{10}} = 83.0$ seconds to compute the K_A_SSI. The function ‘SSI’ from the SFSI R-package offers a functionality for multi-core computing.

### Conclusion

SSIs can be used to optimize prediction accuracy when the training data exhibit complex relationship patterns. In this context, differences in allele frequencies and in LD patterns may make SNP effects heterogenous across families and sub-families, thus making the standard GBLUP sub-optimal. Both local kernels and SSIs can be used to optimize prediction accuracy in such data sets.

## Supplementary information

Supplemental File 1

Supplemental File 2

## Data Availability

Data used in this study is available through the Dryad data repository (10.5061/dryad.qjq2bvqgz). Scripts showing how to perform all the analyses are contained in File S1. All supplemental figures and tables are provided in File S2.
