# Supplementary material for: Multi-generation genomic prediction of maize yield using parametric and non-parametric sparse selection indices
Source: Heredity (Edinb). 2021 Sep 25;127(5):423–32. doi: 10.1038/s41437-021-00474-1 (PMC8551287; doi:10.1038/s41437-021-00474-1)

Supplemental material for Lopez-Cruz *et al.*

In this section, we show how to perform analyses for the multi-generation DH maize data.

Installing the R-packages

The following code can be used to install all needed R-packages

Box 1. Install packages

```
# Install SFSI and BGLR packages
install.packages('SFSI')
install.packages("BGLR")
```

Preparing data

The snippet below shows how to prepare data for GY trait in optimal environment. This information can be changed through the argument `trait`. Phenotypic data is centered and scaled within cycle. Genomic relationship is also calculated. For demonstration purposes, 200 individuals from each cycle will be sampled to run the analyses

Box 2. Prepare data

```
X <- read.csv("Geno_data.csv",row.names = 1)  # Load data
Y <- read.csv("Pheno_data.csv",row.names = 1)

trait <- "GY_Optimal"  # Trait GY, AD, or PH in environment Optimal or Drought

Y <- Y[which(!is.na(Y[,trait])),]
X <- X[rownames(Y),]

if(TRUE){   # set to TRUE to subset data, FALSE otherwise
 nSubset <- 200
 Y <- do.call(rbind,lapply(split(Y,Y$Year),function(x){
     x[sample(1:nrow(x),ifelse(nrow(x)>nSubset,nSubset,nrow(x))),]}))
 rownames(Y) <- unlist(lapply(strsplit(rownames(Y),"\\."),function(x)x[2]))
 X <- X[rownames(Y),]
}

# Center and scale within cycle
Z <- model.matrix(~0+factor(Y$Year))
m0 <- Z %*% tapply(Y[,trait],Y$Year,mean)
sd0 <- Z %*% tapply(Y[,trait],Y$Year,sd)
y <- as.vector((Y[,trait] -m0)/sd0)

# Calculate G matrix
G <- tcrossprod(scale(X))/ncol(X)

save(y,G,Y,trait,file="prepared_data.RData")
```

Obtaining partitions of the prediction cycle

Code below can be used to split the prediction cycle into two independent sets. In this example, the 2020 data (specified by `PSyear = 2020`) is split into 100 different partitions of 15% (specified by `pPS = 0.15`) and (the remaining) 85%. The output is a matrix with 1's (indexing the 85%-set) and 2's (indexing the 15%-set).

Box 3. Get partitions

```
load("prepared_data.RData") # Load data

PSyear <- 2020              # Cycle of prediction
pPS <- 0.15                 # Percentage to split PS (15%-85%)
nParts <- 100               # Number of partitions

iPSyear <- which(Y$Year %in% PSyear)
nPSyear <- length(iPSyear)
nPS <- ceiling(pPS*nPSyear)

# Seeds for randomization
seeds <- round(seq(from=100, to=.Machine$integer.max^0.9, length=nParts))

# Create matrix of 1's and 2's: (1:PS, 2:TS)
partitions <- matrix(1,nrow=nPSyear,ncol=length(seeds))
for(j in 1:length(seeds)){
  set.seed(seeds[j])
  partitions[sample(1:nPSyear,nPS),j] <- 2
}

save(partitions,PSyear,iPSyear,nPSyear, file="partitions.RData")
```

Model specification

The following snippet shows how to define the models to implement (argument `GRM`), the cycles to use as training set (argument `TSyears`), the percentage from the prediction cycle to add to the training set (argument `PS2TS`), and the number of repetitions (given by different partitions) to run. Other arguments can be specified. In this example, the analysis is performed for ease, only for 1 partition for the GBLUP and K_2BLUP models using all training sets combinations, where 15% of the data from 2020 is included in the training sets.

Box 4. Models' definition

```
GRM = c("G","K1","K2","K3","KA")[c(1,3)]
TSyears = c("2017+2018+2019","2018+2019","2019","2018","2017")[c(1:5)]
pPS2TS = c(0,0.05,0.10,0.15)[4]       # Proportion from PS to add to TS
nRep = 1                              # Number of partitions to run
bw = c(K1=0.2,K2=1,K3=5)              # Bandwidth for K1, K2, K3 kernels
nIter = 200;  burnIn = 50            # nIter and burnIn for Bayesian models
burnIn = 50
nCores = 3                           # Number of cores for parallelizing

save(GRM,TSyears,pPS2TS,nRep,bw,file="model_pars.RData")
```

Standard BLUP models

Code below illustrates how to implement the standard BLUP models for all specifications together (training sets, models and number of repetitions) given in **Box 4**.

Box 5. Fit BLUP models

```
library(SFSI); library(BGLR)
load("prepared_data.RData"); load("partitions.RData"); load("model_pars.RData")

OUT1 <- c()    # Output object
for(TS in TSyears)
{
  for(rep in 1:nRep)
  {
    iTS <- which(Y$Year %in% unlist(strsplit(TS,"\\+")))
    iPS <- iPSyear[partitions[,rep]==1]
    iPS2TS <- iPSyear[partitions[,rep]==2][1:ceiling(pPS2TS*nPSyear)]
    if(pPS2TS == 0) iPS2TS <- c()
    iTS <- c(iTS,iPS2TS)
    yNA <- y;   yNA[iPS] <- NA

    index <- c(iTS,iPS)
    y0 <- y[index]; yNA <- yNA[index]
    G0 <- G[index,index]
    D0 <- cov2dist(G0);    D0 <- D0/mean(D0)   # Distance matrix
    ETA <- list(list(K=exp(-bw["K1"]*D0),model="RKHS"),
                list(K=exp(-bw["K2"]*D0),model="RKHS"),
                list(K=exp(-bw["K2"]*D0),model="RKHS"))  # For the KA-BLUP

    for(mm in GRM)         # Running models
    {
      if(mm == "KA"){
        fm0 <- BGLR(yNA,ETA=ETA,burnIn=burnIn,nIter=nIter,verbose=FALSE)
        tmp <- c(fm0$ETA[[1]]$varU, fm0$ETA[[2]]$varU, fm0$ETA[[3]]$varU)
        varUt=paste(tmp,collapse=","); varU=sum(tmp); varE=fm0$varE; mu=fm0$mu
        acc <- cor(fm0$yHat[is.na(yNA)],y0[is.na(yNA)])

      }else{
        if(mm == "G"){
          K0 <- G0
        }else  K0 <- exp(-bw[mm]*D0) # Either K1, K2, K3

        fm0 <- fitBLUP(yNA,K=K0)
        varUt=varU=fm0$varU; varE=fm0$varE;  mu=as.vector(fm0$b)
        acc <- cor(fm0$u[is.na(yNA)],y0[is.na(yNA)])
     }

      tmp=data.frame(mm,rep,length(iTS),varUt,varE,varU/(varU+varE),mu,0,acc)
      names(tmp)=c("GRM","rep","nSUP","varU","varE","h2","mu","lambda","accuracy")
      OUT1=rbind(OUT1,data.frame(TS,nTS=length(iTS),nPS=length(iPS),tmp))
      cat("    TS=",TS,"\t Model=",mm,"-BLUP \t Rep=",rep,"\n")
      cat("    H2=",varU/(varU+varE),"\n")
    }
  }
}

save(OUT1,trait,PSyear,pPS2TS,GRM, file="accuracy_BLUP.RData")
```

Sparse Selection Indices

The following code below shows how to implement the SSI for all specifications together (training sets, models and number of repetitions) given in **Box 4**. This code uses variance components computed in **Box 5** (from the standard BLUP models)

Box 6. Fit SSI models

```
library(SFSI); library(BGLR)
load("prepared_data.RData"); load("partitions.RData");
load("model_pars.RData");    load("accuracy_BLUP.RData")

OUT2 <- c()    # Output object
for(TS in TSyears)
{
  for(rep in 1:nRep)
  {
    iTS <- which(Y$Year %in% unlist(strsplit(TS,"\\+")))
    iPS <- iPSyear[partitions[,rep]==1]
    iPS2TS <- iPSyear[partitions[,rep]==2][1:ceiling(pPS2TS*nPSyear)]
    if(pPS2TS == 0) iPS2TS <- c()
    iTS <- c(iTS,iPS2TS)
    yNA <- y;   yNA[iPS] <- NA

    index <- c(iTS,iPS)
    y0 <- y[index]; yNA <- yNA[index]
    G0 <- G[index,index]
    D0 <- cov2dist(G0);    D0 <- D0/mean(D0)   # Distance matrix
    ETA <- list(list(K=exp(-bw["K1"]*D0),model="RKHS"),
                list(K=exp(-bw["K2"]*D0),model="RKHS"),
                list(K=exp(-bw["K3"]*D0),model="RKHS"))  # For the KA-BLUP

    for(mm in GRM)          # Running models
    {
      OUT0 = OUT1[OUT1$TS==TS & OUT1$GRM==mm & OUT1$rep==rep,]
      varU=OUT0$varU; varE=OUT0$varE; h2=OUT0$h2; mu=OUT0$mu

      if(mm == "KA"){
        aa = as.numeric(unlist(strsplit(varU,",")))
        varU = sum(aa);   aa = aa/sum(aa)
        K0=aa[1]*exp(-bw[1]*D0)+aa[2]*exp(-bw[2]*D0)+aa[3]*exp(-bw[3]*D0)
      }else{
        if(mm == "G"){ K0 <- G0
        }else  K0 <- exp(-bw[mm]*D0) # Either K1, K2, K3
        varU <- as.numeric(varU)
      }

      fmSICV=SSI_CV(y0,K=K0,h2=h2,b=mu,trn=!is.na(yNA),mc.cores=nCores,nFolds=10)
      lambda = summary(fmSICV)$optCOR["mean","lambda"]
      fmSI=SSI(y0,K=K0,h2=h2,b=mu,tst=is.na(yNA),trn=!is.na(yNA),lambda=lambda)
      res = summary(fmSI)

      tmp=data.frame(mm,rep,res$df,varU,varE,h2,mu,lambda,res$acc)
      names(tmp)=c("GRM","rep","nSUP","varU","varE","h2","mu","lambda","accuracy")
      OUT2=rbind(OUT2,data.frame(TS,nTS=length(iTS),nPS=length(iPS),tmp))
      cat("    TS=",TS,"\t Model=",mm,"-SSI\t Rep=",rep,"\n")
    }
  }
}
save(OUT2,trait,PSyear,pPS2TS,GRM, file="accuracy_SSI.RData")
```

Displaying results

The following code shows how to retrieve results from **Box 5** and **Box 6.** The code calculates the average across all repetitions and displays a plot with the results. The results are only for the training sets, models, and number of repetitions specified in **Box 4**.

Box 7. Display results

```
library(ggplot2)
load("accuracy_BLUP.RData");  load("accuracy_SSI.RData")

DAT <- rbind(data.frame(type="BLUP",OUT1),data.frame(type="SSI",OUT2))
DAT$method <- paste0(DAT$GRM,DAT$type)
DAT$method <- factor(DAT$method,levels=unique(DAT$method))
DAT$TS <- factor(DAT$TS,levels=unique(DAT$TS))
DAT <- aggregate(accuracy~TS+method,data=DAT,mean)

ggplot(DAT,aes(TS,accuracy,fill=method)) + theme_bw() +
geom_bar(stat="identity",position=position_dodge(0.8),width=0.8)+
geom_text(aes(y=accuracy*0.9,label=sprintf('%.2f',accuracy)),
                 hjust=1,angle=90,position=position_dodge(0.8)) +
labs(title=paste("Prediction of",PSyear,trait,"\n TS +",100*pPS2TS,"% of",PSyear)) +
theme(plot.title=element_text(hjust=0.5))
```

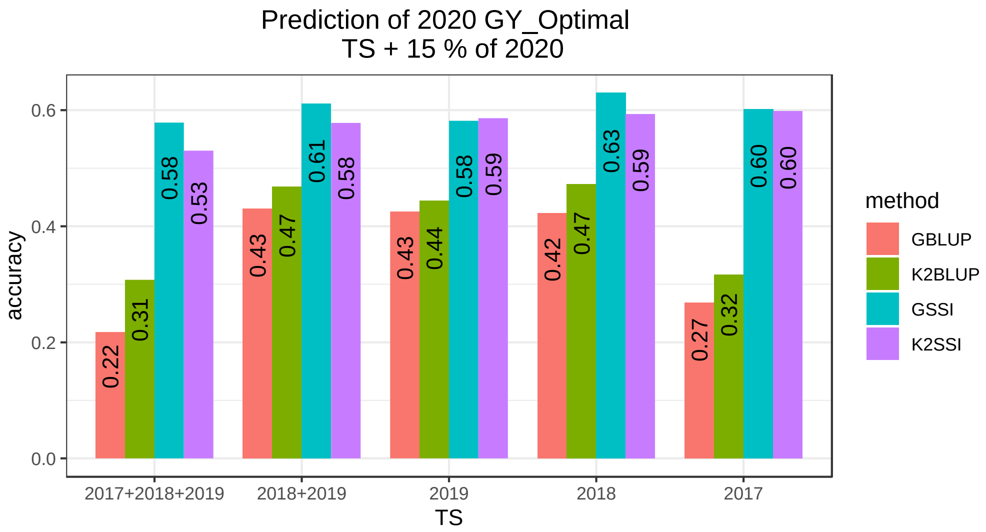

Supplement: Supplementary file 1 — Supplemental File 1 [file 41437_2021_474_MOESM1_ESM.pdf]
