# Supplementary material for: Multi-generation genomic prediction of maize yield using parametric and non-parametric sparse selection indices
Source: Heredity (Edinb). 2021 Sep 25;127(5):423–32. doi: 10.1038/s41437-021-00474-1 (PMC8551287; doi:10.1038/s41437-021-00474-1)
Supplement: Supplementary file 2 — Supplemental File 2 [file 41437_2021_474_MOESM2_ESM.pdf]

Supplemental Material

Multi-generation genomic prediction of maize yield using parametric and non-parametric sparse selection indices

Marco Lopez-Cruz[1,5,*], Yoseph Beyene[2], Manje Gowda[2],

Jose Crossa[3,4], Paulino Pérez-Rodriguez[4], and Gustavo de los Campos[5,6,7]

[1] Department of Plant, Soil and Microbial Sciences, Michigan State University, East Lansing, MI, USA. [2] Global Maize Program, International Maize and Wheat Improvement Center (CIMMYT), Nairobi, Kenya. [3] Biometrics and Statistics Unit, International Maize and Wheat Improvement Center (CIMMYT), Mexico. [4] Colegio de Postgraduados, Montecillos, Edo. de México 56230, Mexico. [5] Department of Epidemiology and Biostatistics, Michigan State University, East Lansing, MI, USA. [6] Department of Statistics and Probability, Michigan State University, East Lansing, MI, USA. [7] Institute for Quantitative Health Science and Engineering, Michigan State University, East Lansing, MI, USA

* Corresponding author. E-mail: lopezcru@msu.edu.

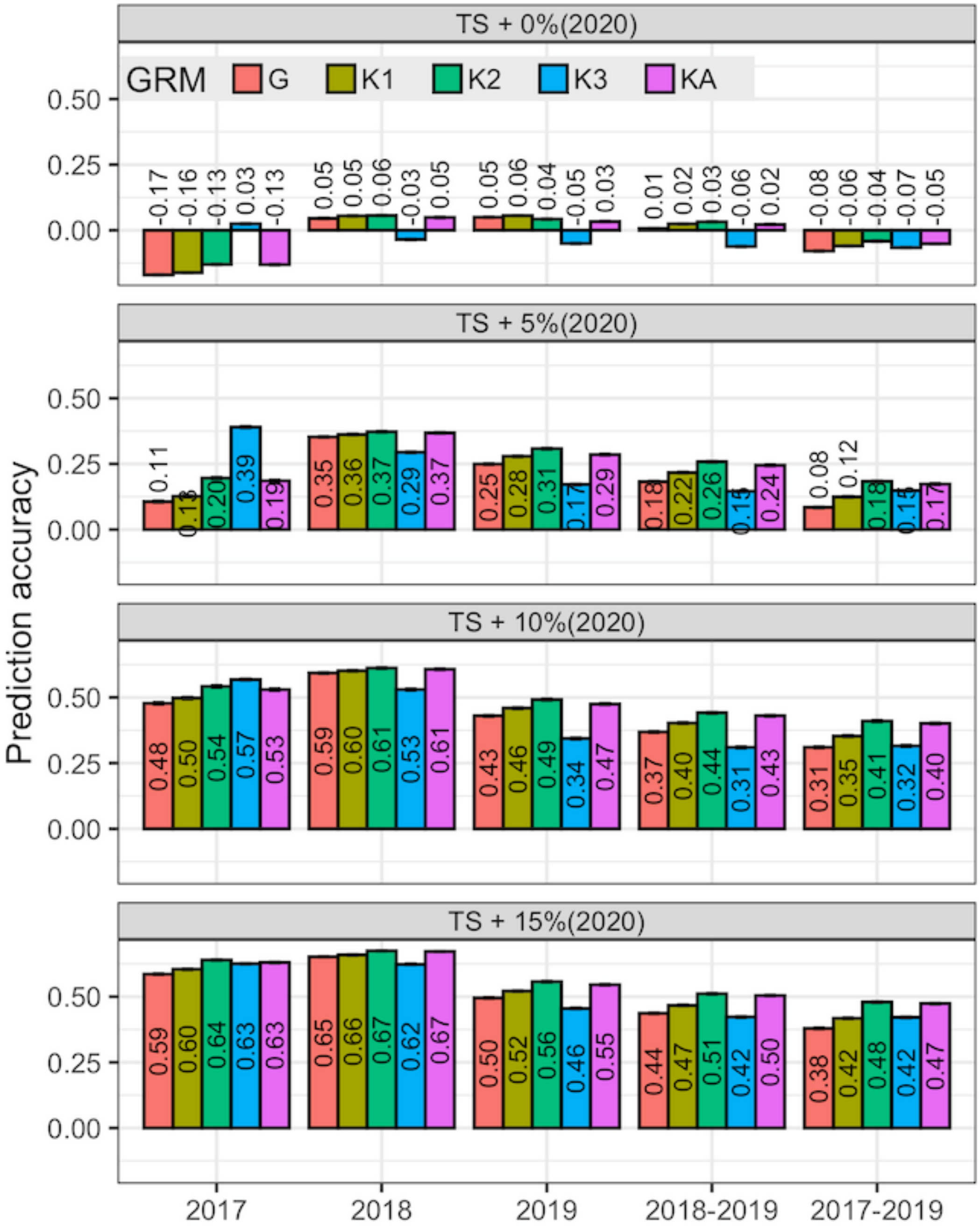

Figure S1. Prediction accuracy of the BLUP models by training set (TS). Models were fitted using different genetic relationship matrices ($\boldsymbol{G}$, $\boldsymbol{K}_1$, $\boldsymbol{K}_2$, $\boldsymbol{K}_3$, or $\boldsymbol{K}_A$). TSs consisted of all the data from the single cycles 2019, 2018, or 2017 alone (top-left panel), or in combination with a proportion (5%=25, 10%=49, 15%=73) of the data from the 2020 cycle. The prediction set consisted of 413 genotypes (representing the 85%) from the 2020 cycle that were not used for model training. Trait GY, drought environment

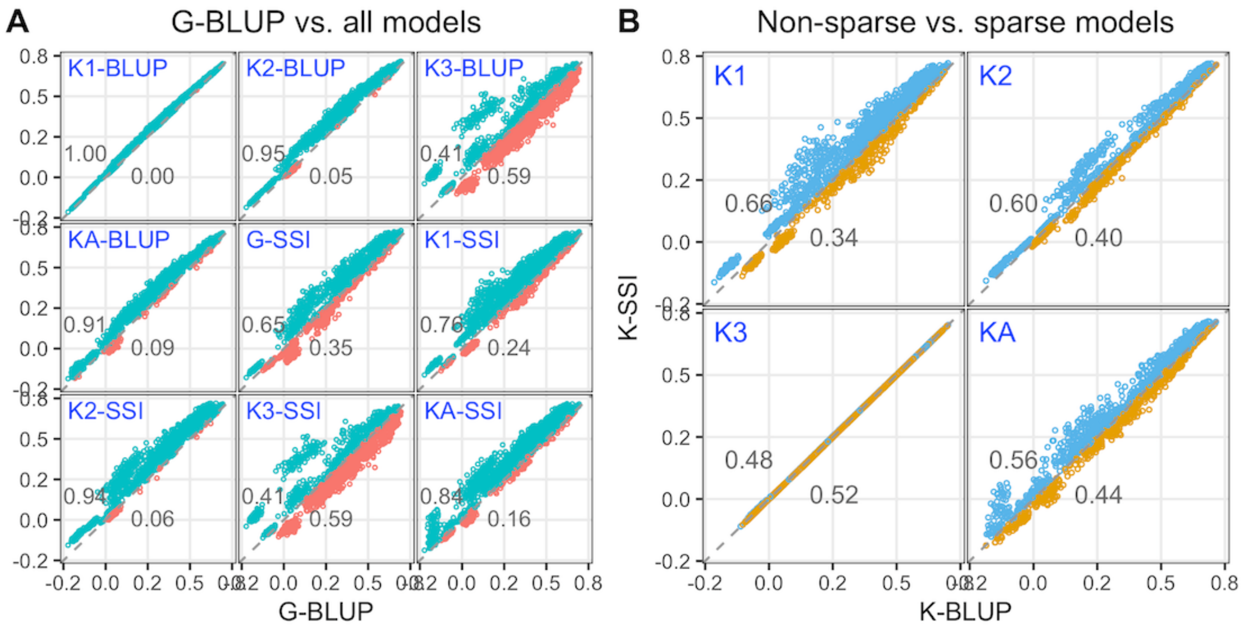

Figure S2. (**A**) Prediction accuracy of the standard (non-sparse) GBLUP model (horizontal axis) versus the prediction accuracy of all other models (vertical axis of each panel). (**B**) Prediction accuracy of the standard KBLUP model (horizontal axis) versus the prediction accuracy of the KSSI (vertical axis) by type of kernel used in panels. Each point represents a training-testing partition within each training set composition. Colored dots (numbers) above (below) the 45-degree line represent cases (the proportion) for which one model outperformed the other model. Trait GY, drought environment

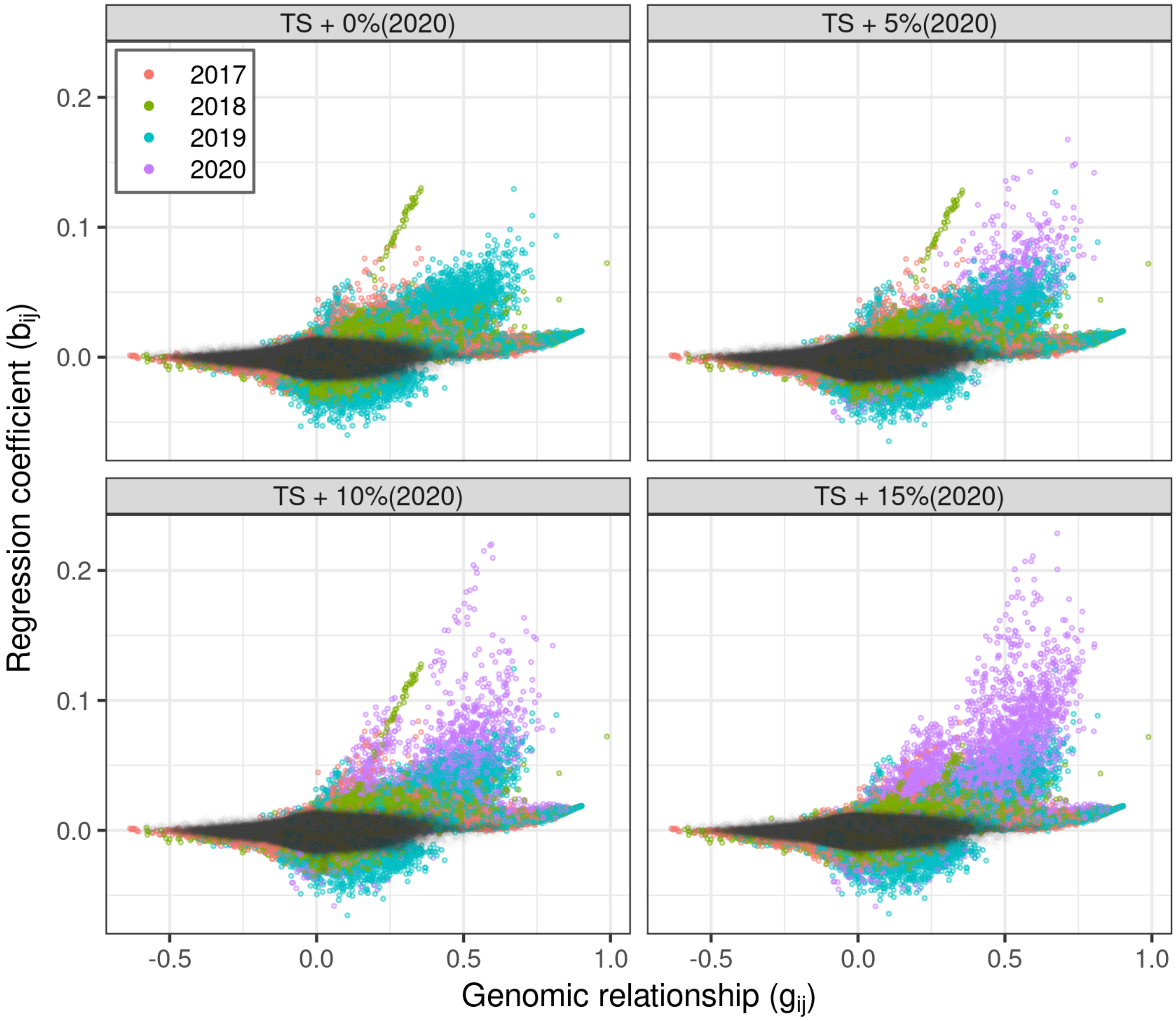

Figure S3. Regression coefficients (b_{ij}) of a standard GBLUP versus the genomic relationship (g_{ij}) for a single training-prediction (TS-PS) partition; i index testing individuals ($i = 1, 2, \ldots, n_{PS} = 413$) and j index training individuals ($j = 1, 2, \ldots, n_{TS}$). The TS is composed by individuals from 2017+2018+2019 cycles alone ($n_{TS} = 3041$, top-left panel) or in combination with a proportion (5%=25, 10%=49, 15%=73) of the 2020 data. Point-color indicates the cycle that training individuals belong to. Grey-color points correspond to individuals whose coefficient is zeroed-out in the GSSI. Trait GY, optimal environment

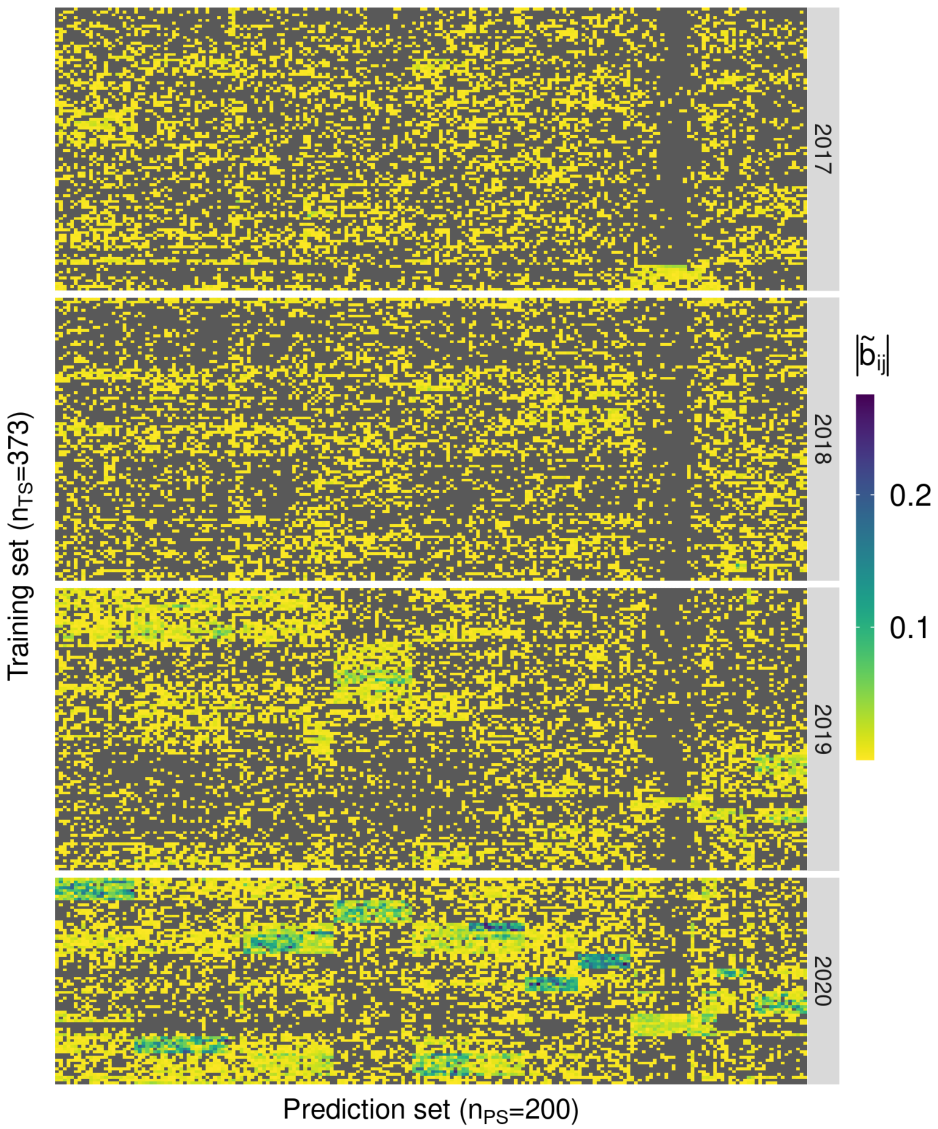

Figure S4. Heatmap of the coefficients in the Hat matrix ($\widetilde{\boldsymbol{B}}_K(\lambda)$) of the K_ASSI model for one training-prediction (TS-PS) partition in the prediction of $n_{PS} = 413$ individuals from 2020 using $n_{TS} = 3114$ individuals (2017+2018+2019 plus 15%=73 genotypes from the 2020 cycle). Columns represent (a sample of 200) predicted individuals and rows represent (a sample of 100 individuals from each cycle 2017-2019 and the 73 subjects from 2020) training individuals, separated by cycle. Each column vector represents values of the vector $\widetilde{\boldsymbol{b}}_{i_K}(\lambda) = \{\tilde{b}_{ij}\}, j = 1, \dots, 3114$ (Eq. 3) using a value of λ obtained by cross-validation. Individuals no contributing to the prediction have a coefficient $\tilde{b}_{ij} = 0$ represented in grey color. Individuals with a non-zero coefficient are shown in a yellow-blue scale. Trait GY, optimal environment

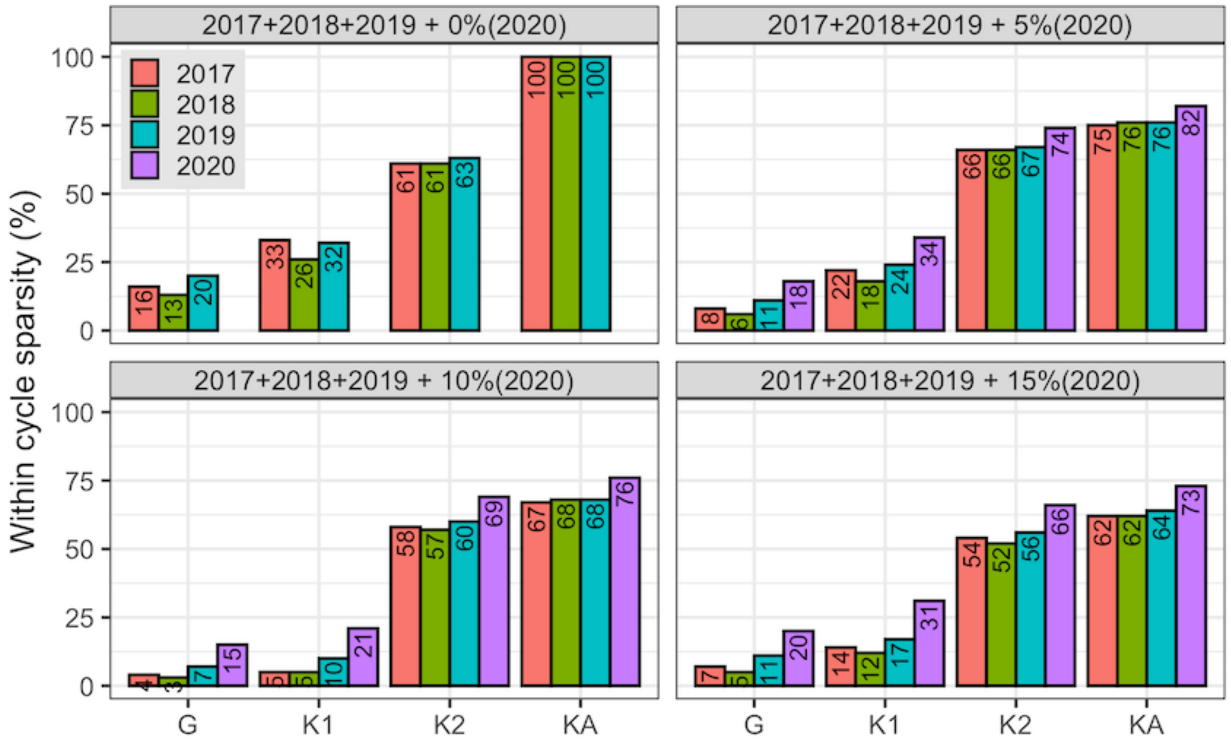

Figure S5. Proportion of the training individuals from each cycle that contributed to the prediction of genotypes from 2020 (averaged across all the 413 testing subjects), using SSIs with different relationship matrices ($\boldsymbol{G}$, $\boldsymbol{K}_1$, $\boldsymbol{K}_2$, or $\boldsymbol{K}_A$). The training set was composed by individuals from 2017 (n = 901), 2018 (n = 1418), and 2019 (n = 722) alone (top-left panel) or in combination with a proportion (5%=25, 10%=49, 15%=73) of the data from the 2020 cycle. Trait GY, drought environment

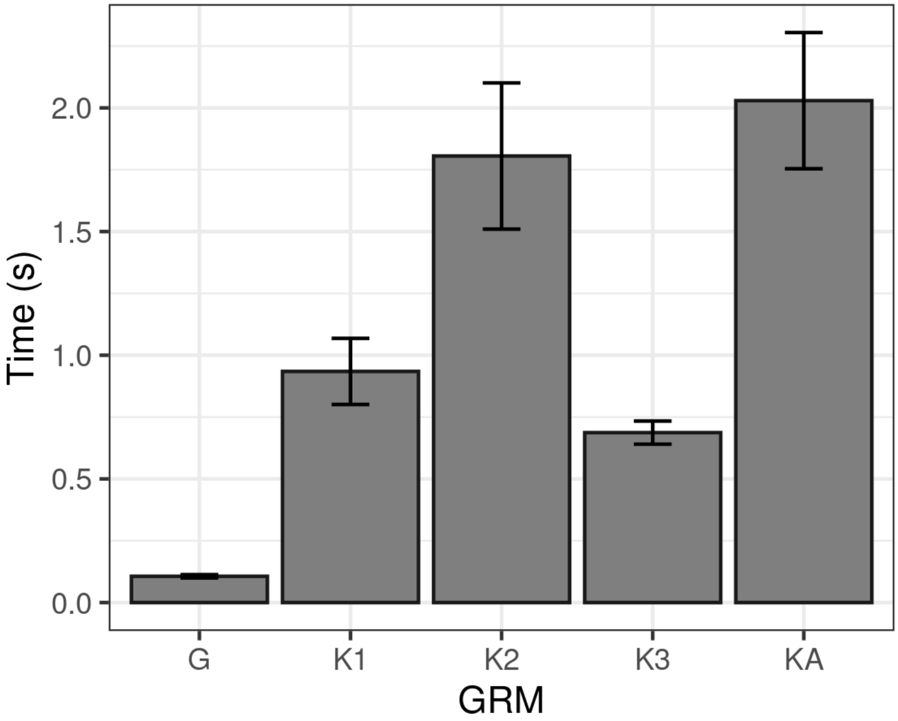

Figure S6. Average time (in seconds) taken by the 'solveEN' function from the SFSI R-package to solve one the penalized problem (Eq. 3) in the prediction of $85\% = 413$ individuals from 2020 using $n_{TS} = 3114$ individuals (2017+2018+2019 plus 15% of the 2020 set), for different genetic relationship matrices ($\boldsymbol{G}$, $\boldsymbol{K}_1$, $\boldsymbol{K}_2$, $\boldsymbol{K}_3$, or $\boldsymbol{K}_A$). The time was averaged across the 413 testing individuals and 100 training-testing partitions. The value of the parameter λ was estimated by cross-validation in the training set within each partition. Trait GY, optimal environment

Table S1. *Accuracy of prediction for each training set (TS) composition (including 0% of subjects from the 2020 cycle), trait GY, optimal environment*

**TS/ n_{TS}**	**GRM**	λ_{CV}[a]	n_{sup}[b] **(%sparsity)**	h^2	**Accuracy (SD)**		**Gain 1 (%)**[c]	**Gain 2 (%)**[d]
					**BLUP**	**SSI**		
2017 n_{TS}=901	G	0.0131	250 (28)	0.50	0.02 (0.019)	-0.03 (0.021)	0	-267
	K1	0.0023	344 (38)	0.86	0.02 (0.019)	0.00 (0.018)	27	-80
	K2	0.0038	369 (41)	0.73	0.04 (0.019)	0.04 (0.020)	131	1
	K3	0.0000	901 (100)	0.96	0.01 (0.018)	0.01 (0.018)	-4	-2
	KA	0.0022	422 (47)	0.85	0.04 (0.024)	0.03 (0.037)	139	-32
2018 n_{TS}=1418	G	0.0059	593 (42)	0.61	0.08 (0.015)	0.15 (0.015)	0	78
	K1	0.0010	736 (52)	0.91	0.09 (0.015)	0.13 (0.015)	11	41
	K2	0.0013	883 (62)	0.79	0.13 (0.015)	0.16 (0.015)	52	26
	K3	0.0000	1418 (100)	0.92	0.13 (0.012)	0.13 (0.013)	60	0
	KA	0.0010	841 (59)	0.88	0.12 (0.020)	0.16 (0.019)	50	29
2019 n_{TS}=722	G	0.0269	97 (13)	0.54	0.18 (0.014)	0.15 (0.013)	0	-18
	K1	0.0070	106 (15)	0.87	0.17 (0.014)	0.14 (0.014)	-7	-17
	K2	0.0053	226 (31)	0.71	0.15 (0.014)	0.15 (0.014)	-18	-1
	K3	0.0000	720 (100)	0.76	0.15 (0.013)	0.15 (0.013)	-16	0
	KA	0.0040	227 (31)	0.80	0.15 (0.017)	0.15 (0.014)	-17	-2
2018-2019 n_{TS}=2140	G	0.0117	394 (18)	0.57	0.17 (0.014)	0.18 (0.012)	0	6
	K1	0.0010	1016 (47)	0.89	0.16 (0.014)	0.17 (0.013)	-8	8
	K2	0.0011	1324 (62)	0.76	0.14 (0.014)	0.15 (0.014)	-18	9
	K3	0.0000	2131 (100)	0.83	0.16 (0.013)	0.16 (0.013)	-6	1
	KA	0.0013	1076 (50)	0.82	0.14 (0.016)	0.16 (0.016)	-16	11
2017-2019 n_{TS}=3041	G	0.0166	360 (12)	0.52	0.12 (0.014)	0.09 (0.014)	0	-26
	K1	0.0031	441 (15)	0.88	0.11 (0.015)	0.14 (0.013)	-11	26
	K2	0.0022	1159 (38)	0.75	0.09 (0.015)	0.11 (0.014)	-23	15
	K3	0.0000	3016 (99)	0.84	0.12 (0.014)	0.12 (0.014)	2	1
	KA	0.0019	1197 (39)	0.80	0.09 (0.017)	0.11 (0.017)	-22	19

GRM: Genetic relationship matrix. SD: standard deviation. h^2: proportion of the trait variance explained by the model. [a]Penalization parameter in Eq. 3 found by cross-validating the TS. [b]n_{sup}=average number of individuals from the TS with a non-zero coefficient in the sparse Hat matrix (support set). %sparsity=$100 \times n_{TS}/n_{sup}$. In the BLUP models, λ_{CV} is equal to zero and n_{sup} is equal to the total TS size. Within each TS cycle, percentage of increase in accuracy of [c]the standard KBLUP relative to the standard GBLUP ($= 100 \times \frac{KBLUP\ accuracy - GBLUP\ accuracy}{GBLUP\ accuracy}$), and of [d]the *SSI relative to the standard *BLUP ($= 100 \times \frac{*SSI\ accuracy - *BLUP\ accuracy}{*BLUP\ accuracy}$, *=G, K_1, K_2, K_3, or K_A)

Table S2. *Accuracy of prediction for each training set (TS) composition (including 5%=25 subjects from the 2020 cycle), trait GY, optimal environment*

TS/ n_{TS}	GRM	λ_{CV}[a]	n_{sup}[b] (%sparsity)	h^2	Accuracy (SD)		Gain 1 (%)[c]	Gain 2 (%)[d]
					BLUP	SSI		
2017 n_{TS}=926	G	0.0158	202 (22)	0.51	0.30 (0.186)	0.32 (0.212)	0	6
	K1	0.0033	239 (26)	0.86	0.31 (0.189)	0.31 (0.186)	4	0
	K2	0.0054	260 (28)	0.74	0.34 (0.192)	0.35 (0.200)	13	1
	K3	0.0000	926 (100)	0.96	0.29 (0.160)	0.29 (0.161)	-4	0
	KA	0.0029	346 (37)	0.85	0.34 (0.189)	0.34 (0.195)	11	0
2018 n_{TS}=1443	G	0.0095	378 (26)	0.61	0.32 (0.127)	0.37 (0.111)	0	16
	K1	0.0017	462 (32)	0.91	0.33 (0.128)	0.38 (0.121)	3	15
	K2	0.0028	558 (39)	0.79	0.35 (0.127)	0.38 (0.118)	9	6
	K3	0.0000	1443 (100)	0.91	0.31 (0.106)	0.31 (0.106)	-4	0
	KA	0.0018	568 (39)	0.88	0.35 (0.125)	0.37 (0.116)	8	7
2019 n_{TS}=747	G	0.0637	31 (4)	0.54	0.34 (0.119)	0.35 (0.149)	0	3
	K1	0.0323	77 (10)	0.87	0.34 (0.124)	0.36 (0.155)	0	4
	K2	0.0259	152 (20)	0.72	0.35 (0.134)	0.35 (0.148)	1	1
	K3	0.0000	731 (98)	0.76	0.31 (0.106)	0.31 (0.106)	-10	0
	KA	0.0234	154 (21)	0.80	0.34 (0.130)	0.35 (0.146)	0	3
2018-2019 n_{TS}=2165	G	0.0179	236 (11)	0.57	0.32 (0.116)	0.36 (0.125)	0	12
	K1	0.0028	356 (16)	0.89	0.33 (0.124)	0.36 (0.130)	1	10
	K2	0.0024	832 (38)	0.76	0.34 (0.135)	0.35 (0.130)	4	5
	K3	0.0000	2165 (100)	0.83	0.31 (0.101)	0.31 (0.101)	-5	0
	KA	0.0019	846 (39)	0.82	0.33 (0.133)	0.35 (0.131)	3	4
2017-2019 n_{TS}=3066	G	0.0175	332 (11)	0.52	0.26 (0.111)	0.28 (0.147)	0	9
	K1	0.0028	484 (16)	0.88	0.27 (0.122)	0.32 (0.124)	2	21
	K2	0.0019	1303 (42)	0.75	0.29 (0.139)	0.31 (0.143)	10	7
	K3	0.0000	3056 (100)	0.84	0.27 (0.110)	0.27 (0.109)	5	0
	KA	0.0015	1424 (46)	0.80	0.28 (0.137)	0.30 (0.141)	9	6

GRM: Genetic relationship matrix. SD: standard deviation. h^2: proportion of the trait variance explained by the model. [a]Penalization parameter in Eq. 3 found by cross-validating the TS. [b]n_{sup}=average number of individuals from the TS with a non-zero coefficient in the sparse Hat matrix (support set). %sparsity=$100 \times n_{TS}/n_{sup}$. In the BLUP models, λ_{CV} is equal to zero and n_{sup} is equal to the total TS size. Within each TS cycle, percentage of increase in accuracy of [c]the standard KBLUP relative to the standard GBLUP ($= 100 \times \frac{KBLUP\ accuracy - GBLUP\ accuracy}{GBLUP\ accuracy}$), and of [d]the *SSI relative to the standard *BLUP ($= 100 \times \frac{*SSI\ accuracy - *BLUP\ accuracy}{*BLUP\ accuracy}$, *=G, K_1, K_2, K_3, or K_A)

Table S3. *Accuracy of prediction for each training set (TS) composition (including 10%=49 subjects from the 2020 cycle), trait GY, optimal environment*

**TS/ n_{TS}**	**GRM**	λ_{CV} [a]	n_{sup} [b] (**%sparsity**)	h^2	**Accuracy (SD)**		**Gain 1 (%)** [c]	**Gain 2 (%)** [d]
					**BLUP**	**SSI**		
2017 n_{TS}=950	G	0.0111	264 (28)	0.53	0.48 (0.083)	0.51 (0.085)	0	6
	K1	0.0016	398 (42)	0.87	0.49 (0.081)	0.52 (0.076)	3	5
	K2	0.0027	413 (43)	0.76	0.52 (0.076)	0.52 (0.078)	8	1
	K3	0.0000	950 (100)	0.95	0.47 (0.087)	0.47 (0.087)	-2	0
	KA	0.0017	461 (49)	0.85	0.51 (0.078)	0.51 (0.083)	7	-1
2018 n_{TS}=1467	G	0.0097	354 (24)	0.61	0.44 (0.074)	0.49 (0.068)	0	10
	K1	0.0016	457 (31)	0.91	0.45 (0.073)	0.49 (0.070)	3	8
	K2	0.0020	683 (47)	0.80	0.48 (0.069)	0.49 (0.068)	9	3
	K3	0.0000	1467 (100)	0.91	0.44 (0.069)	0.44 (0.069)	-1	0
	KA	0.0013	700 (48)	0.88	0.47 (0.068)	0.49 (0.067)	7	3
2019 n_{TS}=771	G	0.0477	59 (8)	0.55	0.46 (0.069)	0.50 (0.068)	0	8
	K1	0.0248	67 (9)	0.88	0.47 (0.069)	0.51 (0.069)	2	10
	K2	0.0083	304 (39)	0.73	0.48 (0.071)	0.49 (0.072)	5	2
	K3	0.0000	763 (99)	0.76	0.43 (0.077)	0.43 (0.077)	-6	0
	KA	0.0091	301 (39)	0.80	0.48 (0.073)	0.49 (0.074)	4	2
2018-2019 n_{TS}=2189	G	0.0139	292 (13)	0.57	0.43 (0.070)	0.48 (0.066)	0	12
	K1	0.0022	447 (20)	0.90	0.44 (0.071)	0.49 (0.066)	3	11
	K2	0.0026	711 (32)	0.77	0.46 (0.071)	0.48 (0.068)	9	4
	K3	0.0000	2189 (100)	0.83	0.42 (0.074)	0.42 (0.074)	-2	0
	KA	0.0019	812 (37)	0.82	0.46 (0.073)	0.48 (0.069)	8	3
2017-2019 n_{TS}=3090	G	0.0171	299 (10)	0.52	0.37 (0.075)	0.43 (0.085)	0	17
	K1	0.0031	396 (13)	0.88	0.39 (0.077)	0.44 (0.081)	5	13
	K2	0.0025	974 (32)	0.75	0.43 (0.080)	0.45 (0.080)	15	6
	K3	0.0000	3088 (100)	0.84	0.40 (0.082)	0.40 (0.082)	7	0
	KA	0.0020	1079 (35)	0.81	0.42 (0.080)	0.44 (0.081)	14	5

GRM: Genetic relationship matrix. SD: standard deviation. h^2: proportion of the trait variance explained by the model. [a]Penalization parameter in Eq. 3 found by cross-validating the TS. [b]n_{sup}=average number of individuals from the TS with a non-zero coefficient in the sparse Hat matrix (support set). %sparsity=$100 \times n_{TS}/n_{sup}$. In the BLUP models, λ_{CV} is equal to zero and n_{sup} is equal to the total TS size. Within each TS cycle, percentage of increase in accuracy of [c]the standard KBLUP relative to the standard GBLUP ($= 100 \times \frac{KBLUP\ accuracy - GBLUP\ accuracy}{GBLUP\ accuracy}$), and of [d]the *SSI relative to the standard *BLUP ($= 100 \times \frac{*SSI\ accuracy - *BLUP\ accuracy}{*BLUP\ accuracy}$, *=G, K_1, K_2, K_3, or K_A)
